# Distribution of local ancestry and evidence of adaptation in admixed populations

**DOI:** 10.1038/s41598-019-50362-2

**Published:** 2019-09-25

**Authors:** Rodrigo Secolin, Alex Mas-Sandoval, Lara R. Arauna, Fábio R. Torres, Tânia K. de Araujo, Marilza L. Santos, Cristiane S. Rocha, Benilton S. Carvalho, Fernando Cendes, Iscia Lopes-Cendes, David Comas

**Affiliations:** 1 Department of Medical Genetics and Genomic Medicine, University of Campinas-UNICAMP, and the Brazilian Institute of Neuroscience and Neurotechnology (BRAINN), Campinas, SP Brazil; 2 Departament de Ciències Experimentals i de la Salut, Institute of Evolutionary Biology (CSIC-UPF), Universitat Pompeu Fabra, Barcelona, Spain; 3 Department of Genetics, Federal University of Rio Grande do Sul, Porto Alegre, RS Brazil; 4 Department of Statistics, Institute of Mathematics, Statistics and Scientific Computing, University of Campinas-UNICAMP, and the Brazilian Institute of Neuroscience and Neurotechnology (BRAINN), Campinas, SP Brazil; 5 Department of Neurology, University of Campinas-UNICAMP, and the Brazilian Institute of Neuroscience and Neurotechnology (BRAINN), Campinas, SP Brazil

**Keywords:** Genetics, Evolutionary biology

## Abstract

Admixed American populations have different global proportions of European, Sub-Saharan African, and Native-American ancestry. However, individuals who display the same global ancestry could exhibit remarkable differences in the distribution of local ancestry blocks. We studied for the first time the distribution of local ancestry across the genome of 264 Brazilian admixed individuals, ascertained within the scope of the Brazilian Initiative on Precision Medicine. We found a decreased proportion of European ancestry together with an excess of Native-American ancestry on chromosome 8p23.1 and showed that this is due to haplotypes created by chromosomal inversion events. Furthermore, Brazilian non-inverted haplotypes were more similar to Native-American haplotypes than to European haplotypes, in contrast to what was found in other American admixed populations. We also identified signals of recent positive selection on chromosome 8p23.1, and one gene within this locus, *PPP1R3B*, is related to glycogenesis and has been associated with an increased risk of type 2 diabetes and obesity. These findings point to a selection event after admixture, which is still not entirely understood in recent admixture events.

## Introduction

Population structure due to genetic ancestry has been a significant confounding factor in genome-wide association studies (GWAS)^1^ since it can potentially lead to results that are not replicated among different populations^2–4^. To date, the majority of GWAS have been performed in European and Asian populations^5,6^ with a poor representation of other geographical areas and admixed populations. Even though ample GWAS reproducibility in medical genetic studies has been claimed^7,8^, it has also been shown that GWAS findings in European populations may not be transferable to admixed American cohorts due to the differences in population substructure, including a specific distribution of ancestry tracts in the genome caused by genetic drift^9^. In addition, admixed populations are likely to hide a larger number of genetic variants that have functional effects^10^, and local ancestry information could aid in identifying causal variants in complex traits^11^. Local ancestry has been inferred in different admixed American individuals, which showed ancestry-specific population substructures and differences in local ancestry proportions^12–18^.

Brazilian individuals encompass recent demographic admixture, including European, sub-Saharan African, and Native-American populations^19–21^. The proportion of global ancestries among the three populations is different between Brazilian and other admixed American populations^13,22,23^ and even among Brazilian individuals from different geographical regions^19,20^. However, local ancestry inference has been poorly explored in Brazilian individuals^20^, and since a specific demographic history can impact human populations differently, their study could potentially add relevant information about the distribution of local ancestry along the human genome. Furthermore, it has been established that ancestry proportions across different regions of the genome could present deviations, which can be due to the lack of methodological power to discriminate between two different ancestries in local ancestry inference, genetic drift after the admixture, or signals of recent natural selection^24–26^.

Therefore, our primary goals are to describe the distribution and to search for possible deviations of local ancestry blocks across the genome of Brazilian admixed individuals. Furthermore, if deviations in local ancestry are found beyond chance, we aim to explore the issue further by searching for signals of recent natural selection and the possibility of association with disease.

## Results

### Population structure and global ancestry inference

**Figure 1:**
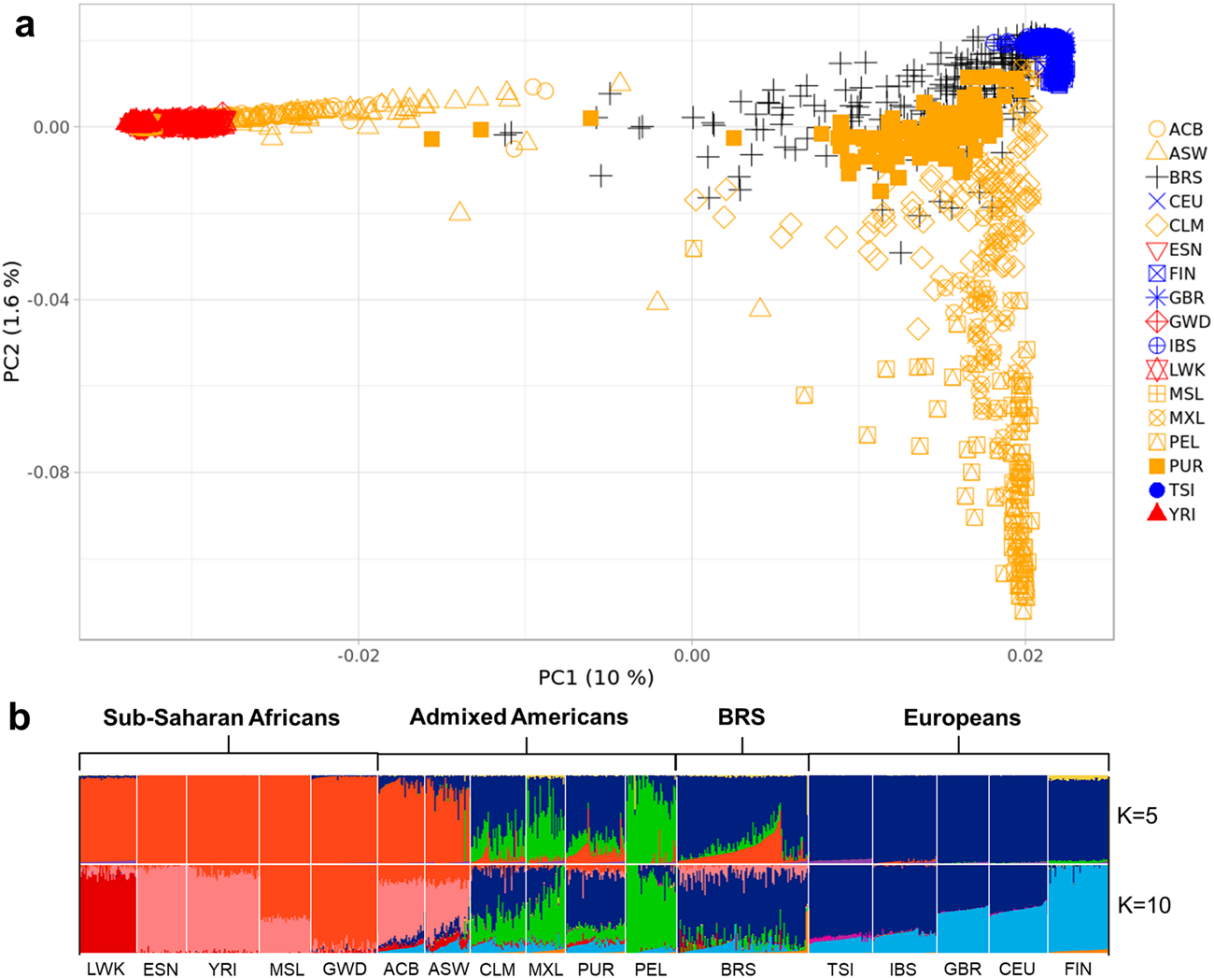
(**a**) The figure depicts a scatterplot showing the two first principal components for the BRS sample (black), Europeans (blue), sub-Saharan Africans (red), and admixed Americans (orange) from 1 KGP. (**b**) Global ancestry inference for ADMIXTURE K = 5 and K = 10, including the BRS and Europeans, sub-Saharan Africans and admixed Americans, where K = 10 had the lowest CV error estimate. Grades of blue, grades of red, and green indicate European, sub-Saharan African, and native American ancestry components, respectively.

In a global PCA with the 1,000 Genomes Project (1 KGP) populations (Fig. 1a and Supplementary Fig. 1), BRS individuals were spread between Europeans, sub-Saharan Africans, and Native-Americans/East Asians, as are other admixed American populations, but were distributed mainly between Europeans and sub-Saharans rather than towards Native-Americans. Therefore, they appeared closer to Puerto Ricans and Colombians than to Peruvians and Mexicans; this is consistent with previous studies^20,22^. We observed similar population distributions when Native-American samples from Mao *et al*.^27^ are included in the PCA plot (Supplementary Fig. 1).

We found the lowest cross-validation (CV) error at K = 10 in the global ancestry inference (0.45247; Supplementary Fig. 2), which shows the separation of subpopulations within continents (Fig. 1b and Supplementary Fig. 3). The component with the highest proportion in the BRS sample was the one most frequent in South Europeans (IBS, TSI; mean = 68.9%; SD = 14.4%), followed by the components most frequent in West Africans (ESN, YRI; mean = 7.7%; SD = 9.2%) and in Native-Americans (mean = 7.6%; SD = 5.8%). We show in the Supplementary Table 1 the remaining ancestry components, including SDs and 95% confidence intervals.

In order to avoid an excess of subpopulation variation in global and local ancestry comparison, we focused on global ancestry inference at K = 5, which identified the ancestry components from the five continental populations presented in the 1 KGP data (Fig. 1b, Supplementary Fig. 3). The BRS sample showed a high ancestry proportion of the European component (blue; mean = 76.9%; SD = 15.7%), followed by sub-Saharan African (red; mean = 13.8%; SD = 11.6%) and Native-American (green; mean = 7%; SD = 5.6%) components (Supplementary Table 1), which is consistent with previous studies^19,20^. We also observed that five individuals located between the European and East Asian clusters in PCA had an East Asian component in the admixture plots (Supplementary Fig. 3). Indeed, after this observation, we were able to retrieve some of the surnames of the volunteers, and four of them bore Japanese surnames.

To evaluate whether the BRS sample presents a geographical substructure based on the state of birth, we performed two tests: the analysis of molecular variance (AMOVA), which compared the genetic distance between the state of birth groups based on a set of 10,000 random SNPs across the genome and separately by chromosome; and  the Kruskal–Wallis test which compared each EUR, AFR, and NAT proportions (ADMIXTURE K = 5) with the state of birth of individuals in the BRS sample. The AMOVA results from the set of 10,000 random SNPs showed that 99.98% of the variation component was observed within groups and 0.02% was observed among groups (total φ-statistics = 0.0002; p-value = 0.2327; Supplementary Table 2), indicating the absence of a population substructure, which is consistent with the lack of clusters observed in the PCA from the BRS sample based on the state of birth (Supplementary Fig. 4). The Kruskal-Wallis test showed EUR p-value = 0.0443, AFR p-value = 0.0567, and NAT p-value = 0.0701, which strengthens the hypothesis of the lack of substructure in the BRS sample based on the state of birth. However, previous studies have found some substructure in Brazilian populations^20,28^. We believe that our results differ from previous reports since most of our samples are from a different geographic region in Brazil, the state of São Paulo. In addition, we based our analysis of population substructure on the state of birth rather than ethnoracial self-classification^28^.

### Local ancestry

Ancestry inference from ADMIXTURE at K = 5 and the average ancestry estimated over all the genome of RFMix from three reference panels, shows a high correlation (Pearson’s correlation ρ > 0.97), indicating consistency between the algorithms (Supplementary Fig. 5). We observed an average local ancestry proportion of 82.7% (SD = 2.2%) across the genome for the European component, 9.8% (SD = 1.3%) for the sub-Saharan African component, and 7.4% (SD = 1.9%) for the Native-American component.

**Figure 2:**
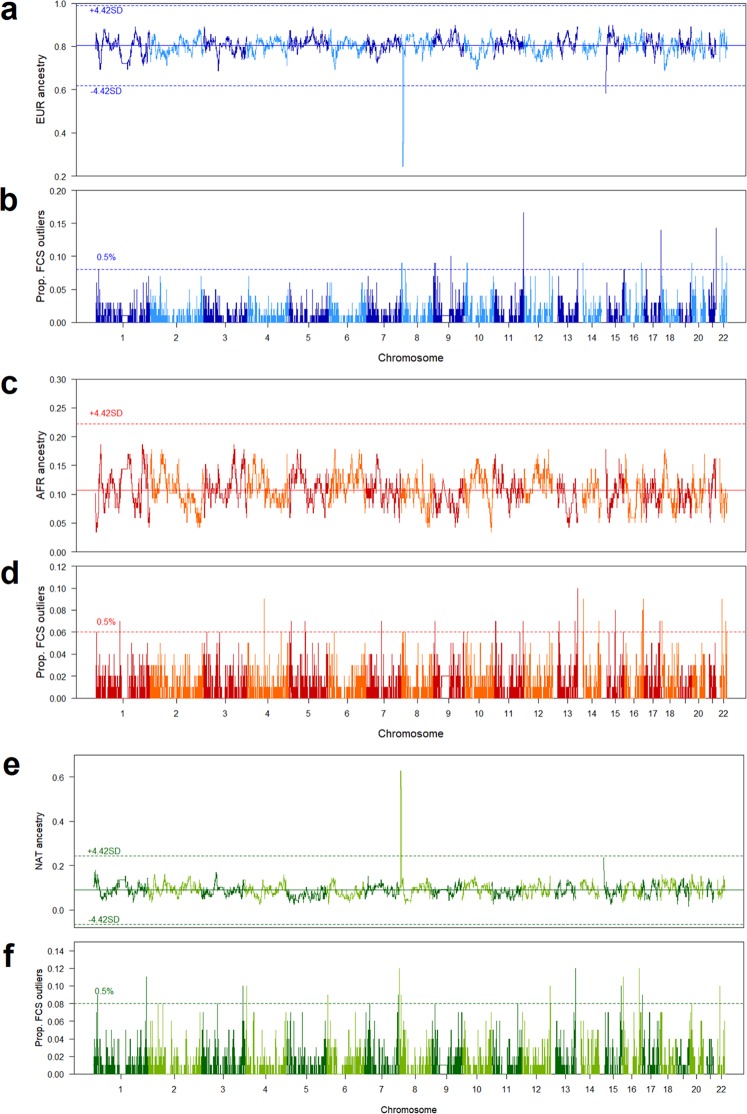
(**a**,**c**,**e**) Local ancestry inference based on the 59 BRS individuals carrying the non-inverted haplotypes (NI/NI), showing each chromosome in the x-axis, and the proportion of European (EUR; grades of blue), sub-Saharan African (AFR; grades of red), and Native-American ancestries (NAT, grades of green) in the y-axis. Each dashed line indicates the 4.42 SD thresholds. (**b**,**d**,**f**) Tests of signals of positive selection based on study design described in Fig. S3, showing the proportion of FCS outliers in the y-axis, following the same colour pattern for each ancestry component. Each dashed line indicates the 99.5^th^ percentile thresholds.

One challenge in our study design was to select an adequate Native-American reference for local ancestry inference. Therefore, we built two different Native-American references, as detailed in the Methods section: the first was based on individuals from the PEL population who presented more than 0.95 of Native-American ancestry proportion at K = 5 in ADMIXTURE, defined here as “Native-American Peruvians” (Supplementary Fig. 6). Based on this approach, we observed a decrease in the European ancestry proportion in the BRS sample on chromosome 8p23.1 (8092025 bp–11859740 bp; Fig. 2a) related to an excess of Native-American ancestry proportion, which was higher than 4.42 SDs from the genome-wide mean (Fig. 2e). The excess of Native-American ancestry in this genetic region was also observed in Colombian, Mexican, and Puerto Rican populations (Supplementary Figs 7–9). However, in a second approach using the pool of Aymaran, Nahuan, Mayan, and Quechuan individuals from Mao *et al*.^27^ as Native-American references, no signal of decreased European or an excess of Native-American ancestry on chromosome 8p23.1 was found in any of the samples, including BRS and Peruvian (Supplementary Figs 7–11).

Chromosome 15cen-q11.2 (20116146 bp–20564575 bp) also showed a decreased European ancestry proportion (Fig. 2a) with an excess of sub-Saharan African (Supplementary Fig. 11) and Native-American Peruvian ancestries (Fig. 2e). Previous studies have shown a low accuracy of local ancestry inference in regions with complex patterns of LD and recombination rates, such as the centromeres, where abrupt deviations from reference panels can lead to a systematic bias in the ancestry inference^24–26^. We found nine SNPs located on the locus at 15cen-q11.2, which covered a region of 448.4 Kb of length divided into 456 tracts in RFMix inference. Among the 456 tracts, up to 260 presented posterior probabilities less than 0.9 (Supplementary Figs 12 and 13). This observation contrasts with what was found on chromosome 8p23.1 (1298 SNPs ranging 3.76 Mb, divided into 575 RFMix tracts), where we found only 76 tracts out of 575 with posterior probabilities less than 0.9 (Supplementary Figs 14 and 15). Therefore, the deviation on local ancestry detected on the locus at 15cen-q11.2 might be due to its location and the low number of SNPs, which could have led to a lack of power of RFMix in local ancestry inference in this genomic region.

### Exploring the 8p23.1 region

**Figure 3:**
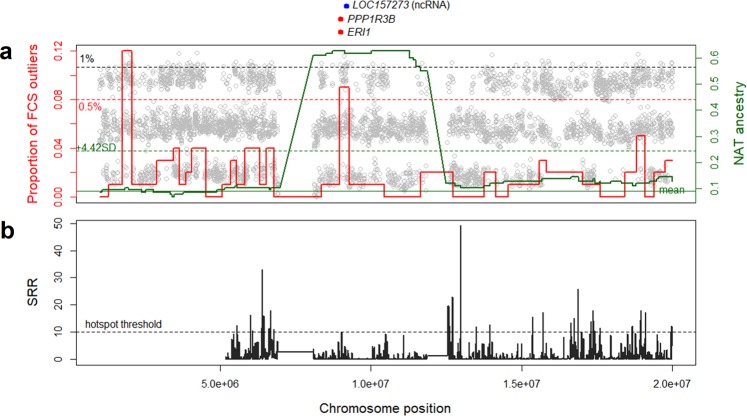
(**a**) Zoom of chromosome 8p23.1, showing the Native-American (NAT) proportion with a solid green line (threshold: dashed green line), and the proportion of FCS outliers with a solid red line (threshold: dashed red line). Each grey dot represents the FCS value of each SNP (threshold: dashed grey line). (**b**) Standardised recombination ratio (SRR) in dark grey. The dashed line shows the threshold for recombination hotspots.

**Figure 4:**
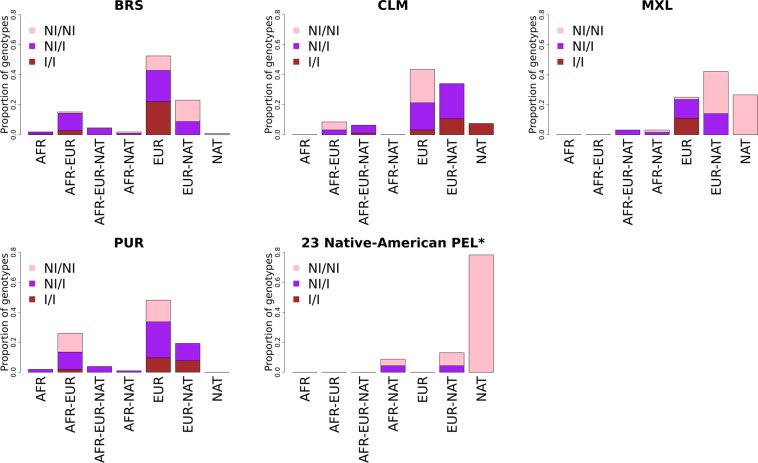
Stacked bar plot showing the proportion of the distribution of NI/NI (pink), NI/I (purple), and I/I (brown) genotypes according to the ancestry tract (x-axis), which was inferred for each admixed American population. The Native-American ancestry tracts from the 23 Peruvians (asterisk) were inferred based on Mao *et al*., 2007 as reference. I: inverted, and NI: non-inverted haplotypes.

To examine the inconsistency of Native-American ancestry estimation when using different references, we studied chromosome 8p23.1 in more detail. We observed that the upstream and downstream flanking regions of the locus on chromosome 8p23.1 were previously described as recombination hotspots and an inversion has been identified in this region (Fig. 3)^29^, which is consistent with the presence of long-range LD haplotypes^30^. Therefore, to discard false-positive results for the excess of Native-American ancestry on 8p23.1 based on inversions, we controlled the local ancestry inferences for the presence (I) or the absence (NI) of the inversion in both the target and reference populations by a haplotype-based method implemented in the *invClust* package in R software^31^. To do that, we analysed the distribution of NI, and I haplotypes on chromosome 8p23.1 in the BRS sample and other admixed American populations and compared NI, and I haplotypes with the ancestry identified on chromosome 8p23.1 (Fig. 4). As shown in Supplementary Table 3, we found 59, 103, and 55 BRS individuals with NI/NI, NI/I, and I/I genotypes, respectively.

The first local ancestry estimations were computed using the entire BRS sample; however, differences in the presence of the non-inversion or inversion segments in each individual could affect the population estimation. Therefore, we classified the BRS and the reference samples as homozygous for the non-inversion (NI/NI) as well as the inversion (I/I). Then, we recalculated the local ancestry distributions and deviations separately. Heterozygous individuals for the inversion (NI/I) were not included because of the inability to perform an accurate identification of the phased chromosome containing the inversion^31,32^. Consequently, it was impossible to match the genotype with the local ancestry estimations. The results show a deviation in local ancestry on chromosome 8p23.1 for the NI/NI genotype group, using both Native-American Peruvians and the pool of Native-American samples from Mao *et al*.^27^ as a reference. We observed an increase in the Native-American ancestry and a decrease in the European ancestry in the 8p23.1 region compared to the genome-wide estimations (Supplementary Fig. 16). We did not observe a deviation in the Native-American ancestry for the I/I genotype group on the 8p23.1 region when using the Native-American individuals from Mao *et al*.^27^ as a reference (we did not find I/I genotypes in the Native-American Peruvian reference). However, we observed a decrease in European ancestry and an increase in sub-Saharan ancestry compared to the genome-wide values in the same genomic region. We performed the same analysis for Colombian, Mexican, and Puerto Rican populations as well as for the 62 admixed Peruvians (using Mao *et al*.^27^ as a reference) (Supplementary Figs 17–20). Interestingly, in the Mexican sample, we observed the same pattern as in the BRS for both the NI/NI and I/I samples, using both Native-American Peruvians and Native-American individuals from Mao *et al*.^27^ as a reference. We also observed the deviation among admixed Peruvians who carry the NI/NI based on the analysis using Native-American individuals from Mao *et al*.^27^ as a reference (Supplementary Fig. 20).

### Haplotype bias evaluation by MDS

However, as stated above, it is important to note that the chromosome 8p23.1 region shows long-range LD haplotypes and is an inverted region, which could affect both the phasing and the local ancestry estimations^30^. Therefore, we performed multidimensional scaling (MDS) analysis based on the IBS values and Hudson’s Fst estimations on chromosome 8p23.1 to verify whether we had any bias in our previous analyses and to provide further proof of ancestry deviation by considering information unbiased by haplotype and LD information. The MDS plot from chromosome 8p23.1 in NI/NI individuals shows three clusters: one for European samples, one for sub-Saharan African samples, and one for Native-American samples (Supplementary Fig. 21a). All BRS NI/NI samples fall in the Native-American cluster, which is consistent with the observations in the local ancestry analyses. The MDS plot for I/I individuals also shows three clusters: one for European samples, one for sub-Saharan African samples, and one for the BRS and MXL individuals (Supplementary Fig. 22a). Unexpectedly, the Native-American individuals fall in the European cluster. These results, together with the fact that BRS and MXL do not cluster with the Europeans, agree with the local ancestry observations. However, the MDS pattern found in the 8p23.1 region could also be found in a set of random genomic regions. To investigate this issue, we estimated MDS among seven genomic regions at random across the genome with the same size of the 8p23.1 region (1883857 bp). The similarity between the MDS plot from the region at chromosome

8p23.1 and each of the MDS plots from the random genomic regions was evaluated by the Procrustes axis rotation analysis. We observed moderate Procrustes correlation values in the NI/NI samples (0.512–0.653; Supplementary Fig. 21b–h) and weak Procrustes correlation values in the I/I genotype (0.271–0.359; Supplementary Fig. 22b–h). Therefore, we suggest that the MDS pattern found in the 8p23.1 locus is not found in a set of random genomic regions.

### Fst estimation on chromosome 8p23.1

Fst estimates on chromosome 8p23.1 show that the NI/NI genotypes from the BRS individuals are more similar to those from Native-American Peruvian and Native-American populations than to those from Sub-Saharan, Colombian, Puerto Rican, and European populations, in agreement with both the local ancestry and the MDS analyses (Supplementary Tables 4 and 6; Supplementary Fig. 23). For the I/I individuals, Fst on chromosome 8p23.1 shows a decreased similarity between BRS and Europeans, and also a decrease between BRS and Native-Americans (Supplementary Tables 5, 6; Supplementary Fig. 24), which it is in agreement with the clusters observed in the MDS. However, the sharp increase in Sub-Saharan African ancestry observed in the local ancestry is not seen in the Fst and is weakly supported by the MDS. This fact could be due to the lack of the appropriate sub-Saharan African proxies (according to slave disembarkation numbers, most sub-Saharan African ancestry in São Paulo and Southeast Brazil is expected to come from West-Central Africa (75%) and Southeast Africa (15%) (http://www.slavevoyages.org) regions, unsampled in the 1 KGP database^33^), or to a false positive in the local ancestry estimation^18^.

In addition, the evaluation of the DNA sequence of chromosome 8p23.1 based on phased haplotypes showed that the DNA sequence from the BRS samples with NI/NI genotype is more similar to the DNA sequence from Native-American PEL samples as reported by Mao *et al*. (2007), than to European and African samples (Supplementary Fig. 25). However, we could not observe the same results for samples with the I/I genotypes, probably due to the reduced number of individuals with the I/I genotype (Supplementary Fig. 26).

Therefore, the NI haplotypes found in the BRS sample are more similar to the NI haplotypes found in non-admixed Native-Americans than the NI haplotypes found in Europeans or some admixed Americans (such as Colombians or Puerto Ricans) although the European genome-wide ancestry found in BRS is much higher than the Native-American ancestry. The conclusions about I/I haplotypes are more challenging, due to the low frequency of the I allele in Native-American populations^34^; however, the lack of shared ancestry with European I haplotypes is clear. Therefore, our results depict a drop in the European ancestry in the region, both in the I and NI haplotypes, which in the case of the NI, is counterbalanced by an increase in the Native-American NI haplotypes.

### Detection of positive selection

Local ancestry proportions more than 4.42 SDs from the genome-wide average could be indicative of positive selection^18,24^ since this deviation is equivalent to a statistical significance p-value < 1 × 10^−5^ from neutrality simulation tests^24^. To check whether the ancestry deviation detected on chromosome 8p23.1 might be related to a positive selection event, we tested our sample for positive selection by five neutrality statistics: intrapopulation absolute integrated haplotype scores (|iHS|, |ΔiHH|)^35,36^, interpopulation integrated haplotype score (|ΔiHH_derived_|)^37^, interpopulation extended haplotype homozygosity (XP-EHH)^36^, and population branch statistics (PBS)^38^ based on Hudson’s Fst^39^.

One tail p-values from the five different statistics were combined using the method proposed by Fisher^18,40^, defined as Fisher combined score (FCS). We assigned as outlier those SNPs within the 1% highest FCS value across the genome. In that way, we found 46 blocks of 100-SNPs with the highest proportion of outlier SNPs: 11 blocks identified as European ancestry; 19 as African; 12 as Native-American; one as European or African; one as African or Native-American; and two as European or Native-American (Supplementary Table 7). Among the Native-American blocks, we found the chromosome 8p23.1 locus (8962666 bp–9265464 bp) (Fig. 2f), which overlaps with the region found with an excess of local ancestry (Fig. 3). However, we did not observe the European and Native-American selection signal on chromosome 8p23.1 when the selection test is conducted separately for NI/NI and I/I individuals, although the selection signal maintains at the threshold line for NI/NI individuals (Supplementary Fig. 16). Although four of these tests are based on extended haplotype homozygosity^35–37^ and the structured haplotype on chromosome 8p23.1 could bias the result, they did not present signals of positive selection, separately. As shown in Fig. 3, the region within chromosome 8p23.1 with increased Native-American component and signals of positive selection spans 320.8 kb and includes two genes, *PPP1R3B* and *ER1*, and one noncoding RNA (*LOC157273*).

## Discussion

Population genetics studies have led to a better understanding of human diseases in the context of human evolution, such as the heterozygous advantage phenomenon in sickle-cell anaemia^41^, cystic fibrosis^42^, and resistance to hepatitis C virus infection^43^. More recently, genome-wide studies have shown different risk allele frequencies from SNPs associated with the disease among populations worldwide^3^, as well as genotypes associated with target treatments in specific populations^44,45^. In this context, local ancestry information across the genome is likely to become relevant to determine whether a genetic variant is expected to be useful in precision medicine, especially for an admixed population. Although local ancestry information is available for admixed American populations^9^, it is still poorly explored in Brazilian individuals^20^. Therefore, in order to fulfil the lack of data, the present study provides information on ancestry tracts across a sample of Brazilian individuals.

Our results showed that the BRS sample is composed of a high proportion of European ancestry followed by sub-Saharan African and Native-American ancestry, showing remarkable variability among BRS individuals. At a genome-wide level, BRS sample is closer to Puerto Ricans and Colombians than to Peruvian and Mexican populations, which is consistent with a large amount of European ancestry detected in other Brazilian samples from different geographic regions^19,20,22,28^. This result is also consistent with previous findings in other admixed American populations, such as in Mexico, Puerto Rico, Peru, Colombia, Ecuador, Chile, and Argentina^13,22,23,46^, including ancestry estimations by different haplotype-based methods^47,48^

Average genome-wide local ancestry showed similar results compared to global ancestry inference in the BRS sample, which indicates consistency between the two ancestry inferences. The local ancestry screening along the genome shows that BRS samples have decreased European ancestry tracts on chromosome 8p23.1, followed by an excess of Native-American ancestry tracts in this same region. This locus is known to present inversion events, which can generate long-range LD regions and could lead to a possible confounding of local ancestry inference^30,49^. Therefore, we further focused our analyses on both homozygous NI/NI and I/I genotype only since we cannot define the ancestry tract for each haplotype from the NI/I individuals. This approach considerably reduced the sample size for analyses performed independently in I and NI individuals.

Brazilian NI/NI samples have extreme lower values of European ancestry in the 8p23.1 region compared to average values along the chromosome 8 and genome-wide. These observations point to the predominance of Native-American origin rather than European of NI haplotypes in the Brazilian population. The Brazilian I/I sample also shows lower values of European ancestry but followed by an increase in sub-Saharan African ancestry. Together, these results point to the presence of selection pressure against the European haplotypes in this genomic region in favour of the Native-American ancestry tracts for the NI haplotypes, and probably the sub-Saharan African tracks for the I haplotypes.

Results in other admixed American populations show variability. Regarding NI/NI individuals, Mexican and Peruvian populations have more similarity on chromosome 8p23.1 with Native-Americans than with European populations, while Colombian and Puerto Rican populations have more similarity with European populations. Therefore, genomic ancestry distribution results cannot be extrapolated to different populations, and differences on 8p23.1 ancestry among populations point to different demographic histories and, more importantly, different selective pressures in the different populations.

Salm *et al*. showed that NI/I and I/I genotypes are rare in Native-American populations due to demographic events^50^, and their study suggests that a serial founder model of migration from Africa explains the demographic distribution of the chromosomal 8p23.1 inversion. We did not find evidence in favour or against this demographic explanation for the I and NI allelic frequency distributions in a pre-Columbian expansion. However, the increase of Native-American component on 8p23.1 in Brazilian, Mexican, and Peruvian samples is difficult to explain by demographic events alone, even considering extreme genetic drift, and it points to a selection event after admixture^24–26^.

Therefore, our findings suggest that either the inversion event occurred more than once, or that genetic drift has produced a high divergence between European and Native-American haplotypes in this genomic region before the European arrival in the Americas. Finally, we suggest that the European NI haplotypes on chromosome 8p23.1 were disadvantaged in favour of the Native-American NI haplotypes in the context of new heterogeneous genomic diversity landscapes and changing environmental conditions which arose after post-Columbian admixture events in the Americas, in Brazil and Mexico. Selection after admixture has been described in archaic admixture between modern humans and Neanderthals^51,52^, but it is not entirely understood in more recent admixture events^53^.

Deviation in local ancestry genomic distribution has also been found on chromosome 6p22 in African-American^14,54^, and on chromosome 8p23.1 in Mexican populations^55^. However, follow-up studies have depicted a critical view of these results, reporting the need for higher thresholds than p > 10^−5^ and the risk of errors in local ancestry inferences^24^, pointing to long-range LD as the possible origin of biased local ancestry predictions^30^. Moreover, they have highlighted the need for parallel analysis with convergent results to have enough evidence of a selection event after an admixture process. Taking these perspectives into account, we have corroborated our local ancestry deviation, reinforcing it with non-haplotype-dependent analyses and selection tests leading to the conclusion of selected Native-American ancestry in the 8p23 region in admixed Brazilians. Therefore, we consider this finding as one of the first pieces of substantial evidence of selection after admixture in admixed Americans, beyond the HLA region^56–58^.

The selection test revealed signs of positive selection on chromosome 8p23.1, in comparison with Peruvians and Native-Americans. Interesting, the *PPP1R3B* gene found within the candidate region encodes a regulatory subunit 3B of the phosphatase-1 protein. *PPP1R3B* plays an essential role in glycogen synthesis, and a previous study showed that *PPP1R3B* overexpression reduces hypoglycaemia after long-term fasting^59^. Indeed, the main clinical phenotypes associated with metabolic disorders, taking admixture into account, are related to type 2 diabetes, insulin secretion, body mass index, obesity, and adiposity^10,59–62^. In this way, we can hypothesise that during the Brazilian population admixture process after 1500 CE, some *PPP1R3B* haplotypes from the Native-American ancestry gene pool could have been advantageous, due to increased fat and glucose storage under a restrictive diet environment, such as severe famine periods^63^, which increased the frequency of the Native-American *PPP1R3B* haplotypes in the Brazilian population. Because of the long-range LD haplotype, due to inversion events, the locus on chromosome 8p23.1 from Native-American ancestry also had the frequency increased by *PPP1R3B* hitchhiking. Future gene expression studies considering genetic ancestry should confirm or disprove this hypothesis.

In conclusion, we found an excess of Native-American haplotypes in the BRS sample at a locus on chromosome 8p23.1, and the presence of different European and Native-American haplotypes, due to long-range LD effects, helped us to understand further the local ancestry deviation. This unusual local ancestry deviation, which could be due to a recent adaptation after the admixture, was further studied, and the hypothesis proposed was supported by additional selection tests. The study of admixed samples allowed us to identify a process of adaptation in a region where the role of selection and demography was unclear. Our results also emphasize the need to study additional admixed American populations independently, avoiding extrapolated conclusions from one to another, since the admixture process can show relevant differences, as we have demonstrated in the present work. Moreover, we propose that individual local ancestry information can be used to obtain relevant information about human health, assisting in the identification of populations that may be at higher risk for specific disorders, which can, in turn, be applied in the recent efforts to develop and implement precision medicine.

## Methods

### Subjects

We analysed 264 healthy individuals (159 females; 105 males) ascertained at the University of Campinas hospital (UNICAMP, Campinas, São Paulo, Brazil) within the scope of the Brazilian Initiative on Precision Medicine Project (BIPMed; http://bipmed.org). We defined them as the Brazilian-São Paulo sample (BRS). Among them, 202 had information about their state of birth (Supplementary Fig. 27). This study was approved by the Research Ethics Committee of the University of Campinas (UNICAMP, Campinas, São Paulo, Brazil), and we obtained informed consent from all participants who signed a consent form before entering the study. All methods were performed in accordance with the relevant guidelines and regulations.

We compared the BRS sample with two publicly available datasets: the first included sequencing data from 2504 unrelated individuals from 26 worldwide populations from the 1000 Genomes Project phase 3 (1 KGP)^33^ (GRCh37/hg19 assembly; ftp://ftp.1000genomes.ebi.ac.uk/vol1/ftp/release/20130502/) (Supplementary Table 8), and the second including genotyped data from 43 Native-American individuals (25 Aymarans, 10 Nahuans, 6 Mayans, and 2 Quechuans) previously studied by Mao *et al*.^27^ and Martin *et al*.^9^.

### SNP genotyping and initial data processing

The genomic DNA of the BRS sample was obtained from peripheral blood using the phenol-chloroform procedure^64^. The quantification and quality of each DNA sample were evaluated by Qubit^®^ 2.0 Fluorometer (Invitrogen, Carlsbad, CA, USA) and Epoch 2 microplate spectrophotometer (BioTek Instruments Inc., Winooski, VT, USA). Genotype calling was performed by Genome-Wide Human SNP Array 6.0 platform (Affymetrix Inc, Santa Clara, CA) in the Laboratory of Multiuser Equipment facility at UNICAMP. We called the genotypes from fluorescent signals by CLRMM algorithm^65^, which were converted to PLINK format^66^ by in-house scripts.

We used PLINK 1.9^66^ software to perform SNP and individual filtering.^67^ Among the 906,600 genotyped SNPs, we removed 1,428 unmapped, 37,149 non-autosomal, 134,006 ambiguous (SNPs with G/C or A/T alleles), and 128,380 SNPs in Hardy–Weinberg disequilibrium (p-value < 0.01)^67^. We also checked for SNP and individual missing data > 2%, but all SNPs and individuals passed this filter because the genotype call rate by the CRLMM algorithm was 100%^65^. After genotype filtering, 605,637 SNPs remained (Supplementary Table 9). We performed the same SNP filtering procedure in the Native-American sample, and 719,472 SNPs remained (Supplementary Table 9).

Since we analysed an admixed population, an excess of heterozygote SNPs could be due to recent population substructure, and SNPs with Hardy-Weinberg disequilibrium could be maintained^23^. However, Hardy-Weinberg disequilibrium filtering is performed to remove possible genotyping errors^67^ and, although it can eliminate signals of selection; it reduces the possibility of finding false-positive results. For this reason, we aimed to be conservative in terms of excluding genotyping errors, including a high p-value threshold, which guarantees that the signals of selection we found are not produced by systematic genotyping errors.

We evaluated the heterozygosity rate for each individual to search for inbreeding (low heterozygosity rates) or sample contamination (high heterozygosity rates)^67^. The mean heterozygosity rate of the BRS sample was 0.317 (SD = 0.012). We removed 11 individuals with heterozygosity rate higher (n = 5) or lower (n = 6) than two SDs from the mean. We also removed eight individuals from pairs of individuals who presented identical-by-state alleles > 0.85, which could indicate duplicated samples^66,67^. Next, we excluded individuals from identical-by-descent and genomic relatedness matrix estimations > 0.125, which is the expected genomic relatedness for third-degree relatives. To maximise the sample size, we used a greedy algorithm implemented in PLINK 1.9 software. After individual filtering, 222 individuals remained suitable for further analysis (Supplementary Table 10). We did not perform individual filtering in the Native-American sample in order to analyse the same 43 individuals evaluated previously by Martin *et al*.^9^.

### Merging of datasets

First, we merged BRS with 1 KGP, removing 5750 SNPs that did not match between the two datasets. After merging, we removed 3,612 SNPs with a minor allele frequency (MAF) < 0.01. To avoid bias in population structure, MDS, and global ancestry inference, we removed 291,734 SNPs in linkage disequilibrium (LD) by the following parameters: window size = 50 SNPs, shift step = 5 SNPs, and r^2^ = 0.5^67^. In the end, we had 304,541 SNPs for the population structure and global ancestry inference and 596,275 SNPs for local ancestry inference (Supplementary Table 9). Next, we generated a second dataset by merging the Native-American sample with the BRS + 1 KGP dataset, and we kept 605,637 SNPs common to both datasets. After MAF and LD pruning, 294,823 SNPs remained for the population structure and global ancestry inference, and 570,323 SNPs remained for local ancestry inference (Supplementary Table 9). The merging process and the following filters were performed by PLINK 1.9 software.

### Population structure and global ancestry inference

We compared the BRS sample and the 26 populations from 1 KGP using principal component analysis (PCA) in PLINK v1.9 software. Global ancestry was inferred by unsupervised ADMIXTURE maximum likelihood approach^68^ from K = 2 to K = 13 ancestral components. We also performed three different runs with different random seeds for each K to observe the consistency of estimates. The BRS sample has a sample size two times larger than other 1 KGP populations, which could lead to bias in admixture results^69^; therefore, we divided the BRS sample into two random subsamples (n = 111) and ran ADMIXTURE separately for each subsample.

### Analysis of molecular variance

Brazilian individuals could present different ancestries based on their geographical location. Therefore, we evaluated possible clustering based on available state of birth information by PCA. In addition, we evaluated whether the state of birth groups are genetically similar by the Analysis of Molecular Variance (AMOVA) using *poppr.amova*^70^ and Kruskal-Wallis test based on EUR, AFR, and NAT ancestry proportions (ADMIXTURE K = 5). Kruskal-Wallis p-values were corrected by Bonferroni adjustment under three comparisons. The analyses were performed by an in-house script developed in R software.

### Local ancestry inference

We phased the SNPs without LD pruning with SHAPEIT2 v2.r387 software using the default parameters^71^. After phasing, we converted the output data from SHAPEIT2 to the input files required by RFMix v.1.5.4 software^32^, following a pipeline reported by Martin *et al*.^9^ (https://github.com/armartin/ancestry_pipeline).

We estimated the Native-American local ancestry from two approaches. The first approach was to build a reference by selecting 23 individuals from the PEL population (Peruvian individuals from 1 KGP) who presented more than 0.95 of Native-American ancestry proportion at K = 5 in ADMIXTURE (18 individuals had > 0.99 Native-American ancestries). To avoid biases due to unbalanced reference panel sizes^32^, we selected 23 random individuals among Europeans (CEU, GBR, IBS, and TSI) and 23 among Africans (ESN, GWD, LWK, MSL, and YRI) (Supplementary Fig. 6a). The second approach was to build the Native-American reference based on the 43 Native-American samples from Mao *et al*.^27^ (25 Aymarans, 10 Nahuans, 6 Mayans, and 2 Quechuans), 43 random individuals among Europeans, and 43 among Africans to evaluate the consistency of local ancestry inference using the first approach (Supplementary Fig. 6b).

We ran RFMix in the PopPhased mode with a minimum window size of 0.2 cM, one EM iteration, and node size 5, maintaining the reference panel after the initial inference step and saving the forwards–backwards probabilities. We developed an in-house R script (https://www.r-project.org/) to analyse the proportion of European, African, or Native-American ancestry among BRS individuals for each SNP across the genome. We inferred local ancestry and estimated the proportion of SNPs associated with each ancestry across the genome for Colombian (CLM), Mexican (MXL), and Puerto Rican (PUR) populations from 1 KGP to compare results with the BRS sample. We also analysed the local ancestry of all PEL individuals in the second local ancestry approach, where we used Native-American individuals from Mao *et al*.^27^ as a Native-American reference.

### Identification of haplotype inversions

Since our results show evidence of haplotype inversions, we identified non-inverted (NI) and inverted (I) haplotypes by a haplotype-based method developed by Cáceres and González (*invClust* package in R)^31^. We set *invClust* parameters as follows: two multidimensional scaling components; method = 1, which performed an EM algorithm for three genotypes; 1,000,000 iterations for the EM algorithm; and 10e^−6^ for EM convergence tolerance.

### Fst estimation and multidimensional scaling analysis

In order to evaluate which haplotype from the 1 KGP populations has a high similarity with the BRS sample, we separated the NI/NI and I/I genotypes from all populations and estimated Hudson’s Fst^39,72,73^ across all chromosome 8 by a 1 Mb window. To complement the Fst information, we calculated and plotted multidimensional scaling (MDS) values from IBS estimations for each individual, using the R software, in the 8p23.1 and additional seven genomic regions at random across the genome with the same size of the 8p23.1 region (1883857 bp). To evaluate the similarity between the MDS plot from 8p23.1 and each MDS plot from the random genomic region, we performed Procrustes axis rotation. We compared the Procrustes results from the MDS plots by estimating the Procrustes correlation test under 10,000 permutations. All Procrustes analyses were performed by the *vegan* package in R^74^.

### Haplotype analysis by DNA sequence

The SHAPEIT software was used to convert the phased haplotypes to VCF files, separating individuals with the NI/NI and the I/I genotype and by continental populations. The VCF files were converted to DNA sequence by *read.vcfR* and *vcfR2DNAbin* from the *vcfR* R package^75^. The DNA sequences were plotted by *image.DNAbin* function from the *ape* R package^76^.

### Positive selection test

We tested our sample for positive selection by five neutrality statistics: intrapopulation absolute integrated haplotype scores (|iHS|, |ΔiHH|)^35,36^, interpopulation integrated haplotype score (|ΔiHH_derived_|)^37^, interpopulation extended haplotype homozygosity (XP-EHH)^36^, and population branch statistics (PBS)^38^ based on Hudson’s Fst^39^. Integrated haplotype scores and extended haplotype homozygosity were estimated by *rehh* package in R software^35,36,57^. PBS and Hudson’s Fst were calculated by an in-house script in R software based on previously reported formulae^38,39^. Interpopulation statistics require a reference population and PBS statistics for an outgroup population. Therefore, the sample we used as a reference population for the scans of positive selection in the BRS group were 404 European (CEU, GBR, IBS, TSI), 504 sub-Saharan African (ESN, GWD, LWK, MSL, YRI), and the 23 Native-American Peruvian (PEL) individuals of the 1 KGP for the first local ancestry approach, and the 43 Native-American individuals^27^ for the second local ancestry approach. PBS was evaluated using 96 Punjabi from Lahore, Pakistan (PJL) as outgroup population. We performed the same positive selection test for Colombian, Mexican, and Puerto Rican populations, to compare results with our sample, and also changed the Native-American reference panel (Supplementary Fig. 28).

The ancestral and derived allele assignation followed the alignment from 1 KGP data. We combined the one tail p-values of the different statistics by the method proposed by Fisher^18,40^ and showed in the following formulae:

$$FCS={\sum }_{i=1}^{k}-lo{g}_{10}({p}_{i}),$$

where *FCS* is Fisher combined score, *k* is the number of combined statistics, and *p*_*i*_ the empirical p-value, i.e., the genomic rank of the i^*th*^ statistics divided by the total number of SNPs^18,40^. Since small values of p_*i*_ lead to large FCS values, we defined outlier SNPs as those with values of FCS higher than 99% of the SNP FCS values across the genome (i.e., the 1% highest FCS values). To search for regions with a signal of positive selection, we split the genome into 100-SNP blocks. Then, we estimated the proportion of outliers within each block, and if a block was found to present a proportion of outliers higher than the 99.5^*th*^ percentile (the highest 0.5%) among the proportions of all blocks, it was defined as a region under positive selection^18,40^. Although FCS violates the assumption of dependency among empirical p-values, it has a chi-square distribution with 2*k* degrees of freedom under neutrality^40^. Previous studies have also shown that combining different neutrality tests increases the power to detect signals of positive selection^37,40^.

## Supplementary information

Supplementary information
