# Supplementary material for: Distribution of local ancestry and evidence of adaptation in admixed populations
Source: Sci Rep. 2019 Sep 25;9:13900. doi: 10.1038/s41598-019-50362-2 (PMC6761108; doi:10.1038/s41598-019-50362-2)
Supplement: Supplementary file 1 — Supplementary information [file 41598_2019_50362_MOESM1_ESM.docx]

Supplementary information

**Distribution of local ancestry and evidence of adaptation in admixed populations**

Rodrigo Secolin, Alex Mas-Sandoval, Lara R. Arauna, Cristiane C. Rocha, Benilton S. Carvalho, Fábio R. Torres, Tânia K. de Araujo, Marilza L. Santos, Fernando Cendes, Iscia Lopes-Cendes, David Comas


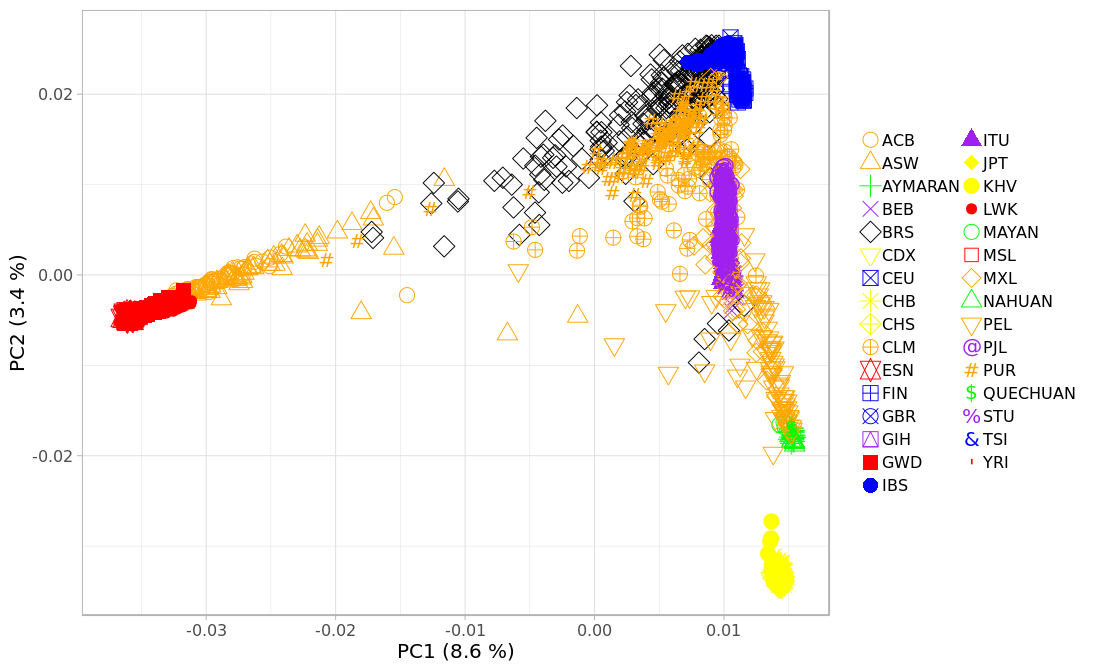


Supplementary Fig. 1. PCA plot showing the BRS sample (black), populations from the 1KGP dataset, and the Native-American individuals from Mao et al, 2007 (green). Each symbol represents a population, and the colors represent the super-populations (red = Africa; blue = Europe; yellow = East Asia; purple = South Asian; orange = admixed American populations).


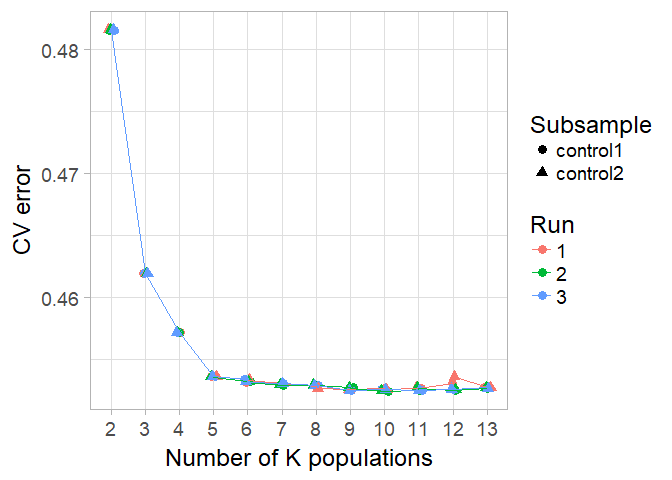


Supplementary Fig. 2. Cross validation (CV) error for K = 2 to K = 13 from SNP array data. Each point represents one run with a different seed. The symbols represent the subsamples and the colours represent the different runs.


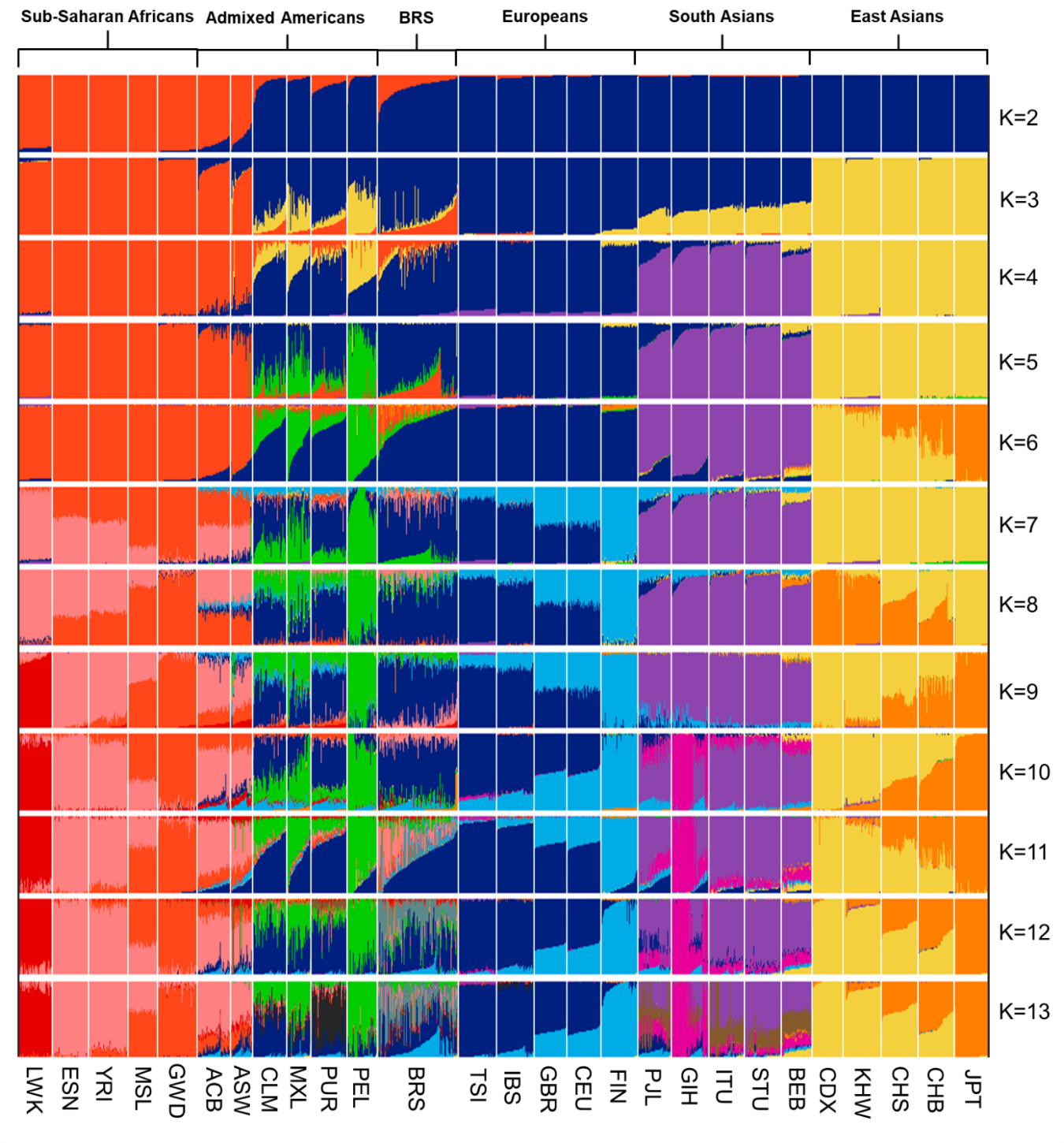


Supplementary Fig. 3. Barplots of admixture proportions from K=2 to K=13. Each barplot represents the lowest CV error among three different runs with different seeds for each K estimation, with K = 10 showing the lowest CV error.


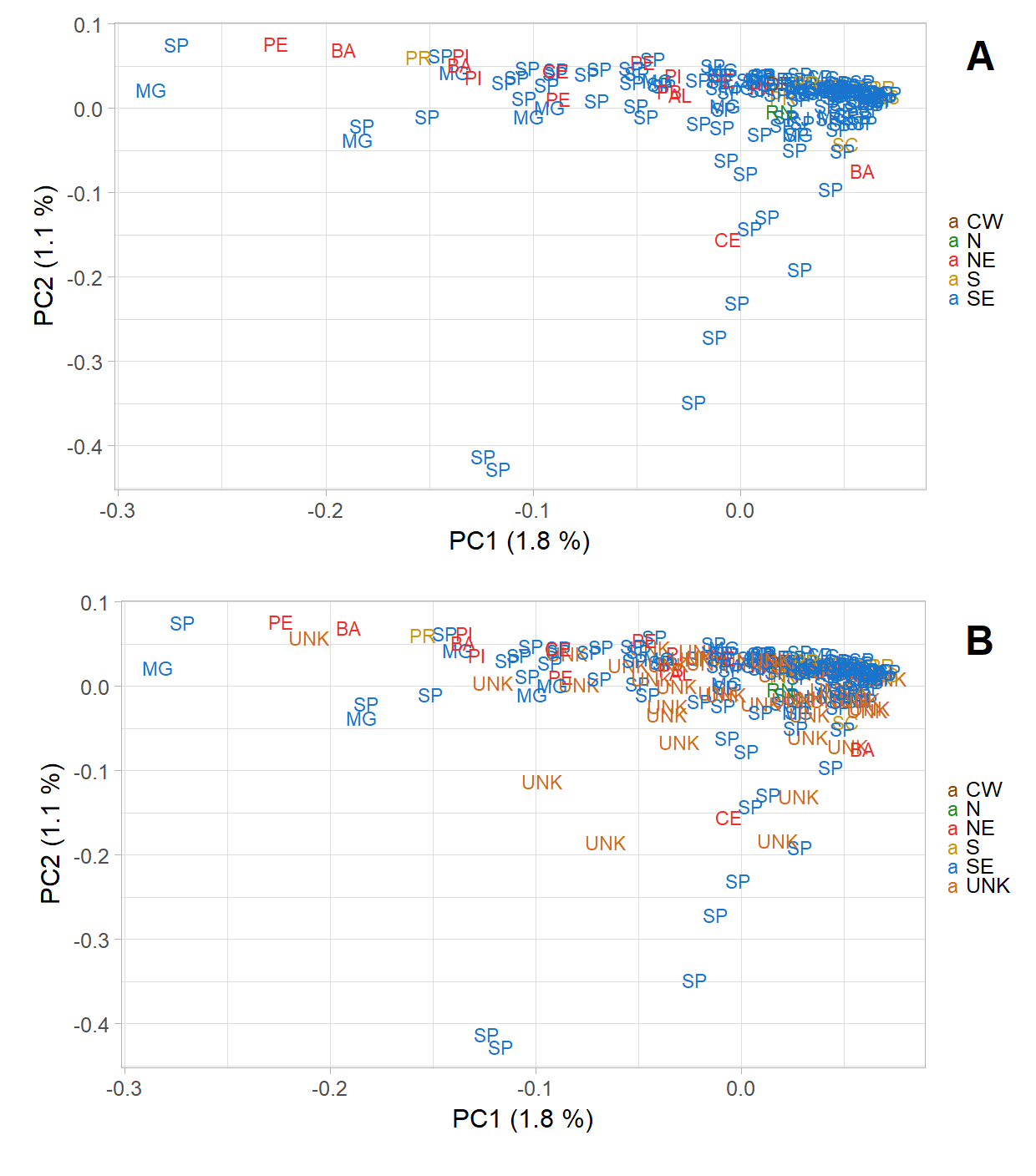


Supplementary Fig. 4. PCA plot of the BRS sample labelled according to the birthplace of the BRS volunteers. (A) Only individuals with state of birth information, and (B) all BRS individuals (including those for whom no state of birth information was available). The percentage of variability of each principal component is showed in each respective axis. Abbreviation indicates each Brazilian state, as the following: AL = Alagoas; BA = Bahia; CE = Ceará; DF = Distrito Federal; GO = Goiás; MG = Minas Gerais; MS = Mato Grosso do Sul; PA = Pará; PB = Paraíba; PE = Pernambuco; PI = Piauí; PR = Paraná; RJ = Rio de Janeiro; RN = Rio Grande do Norte; RS = Rio Grande do Sul; SC = Santa Catarina; SP = São Paulo. Each colour represents one Brazilian geographical region (CW = Centre-West; N = North; NE = Northeast; S = South; SE = Southeast; UNK = unknown).

Supplementary Fig. 5. Correlation plots between ADMIXTURE at K = 5 versus RFMix inferences for EUR, AFR, and NAT ancestries. The lines indicate the best fit of the data by local regression (LOESS) with 95% confidence interval (grey area).


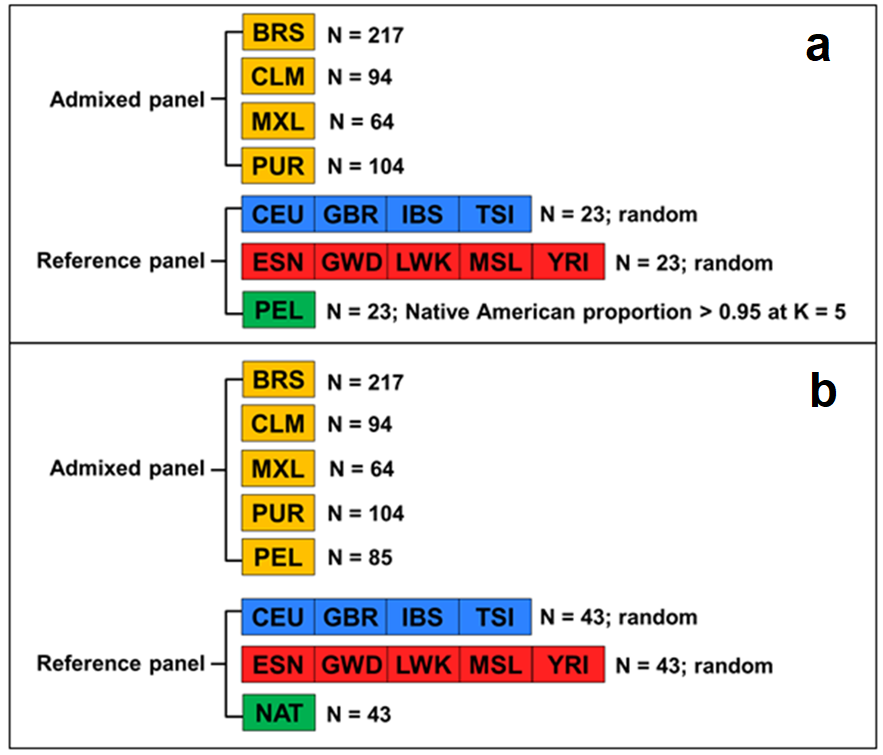


Supplementary Fig. 6. Local ancestry study design. Reference panel included Europeans (blue squares), Africans (red squares), and the Peruvian individuals as Native-American reference (green square) in the first approach (a); and 43 Native-Americans from Mao et al, 2007, in the second approach (b), which allowed us to include the Peruvians in the local ancestry inference. We removed from the analysis the five BRS individuals with Japanese ancestry, shown in Figure 1.


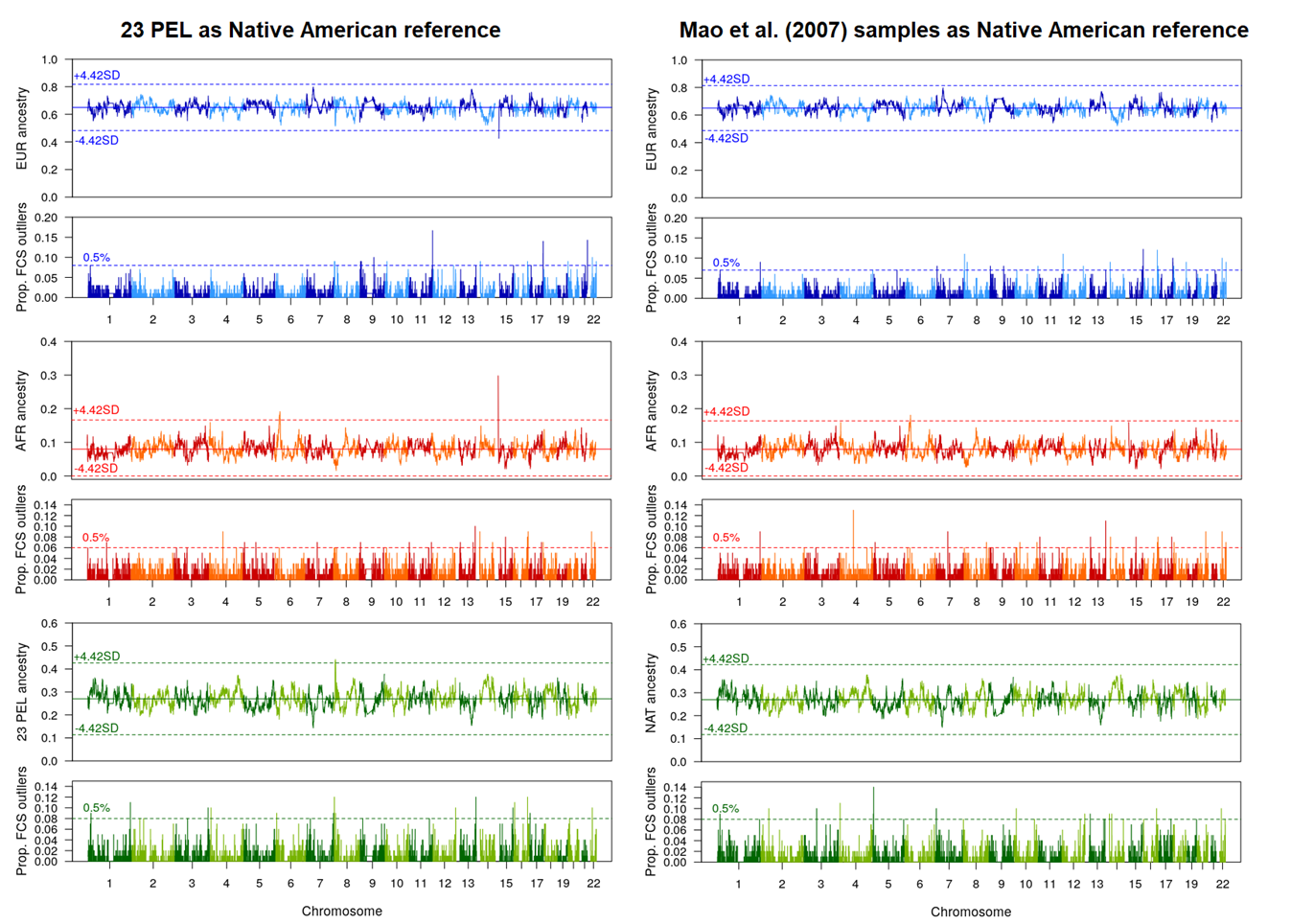


Supplementary Fig. 7. CLM population local ancestry estimation and test of natural selection. We based on NAT ancestry from the 23 PEL individuals and from the 43 individuals from Mao et al., 2007.


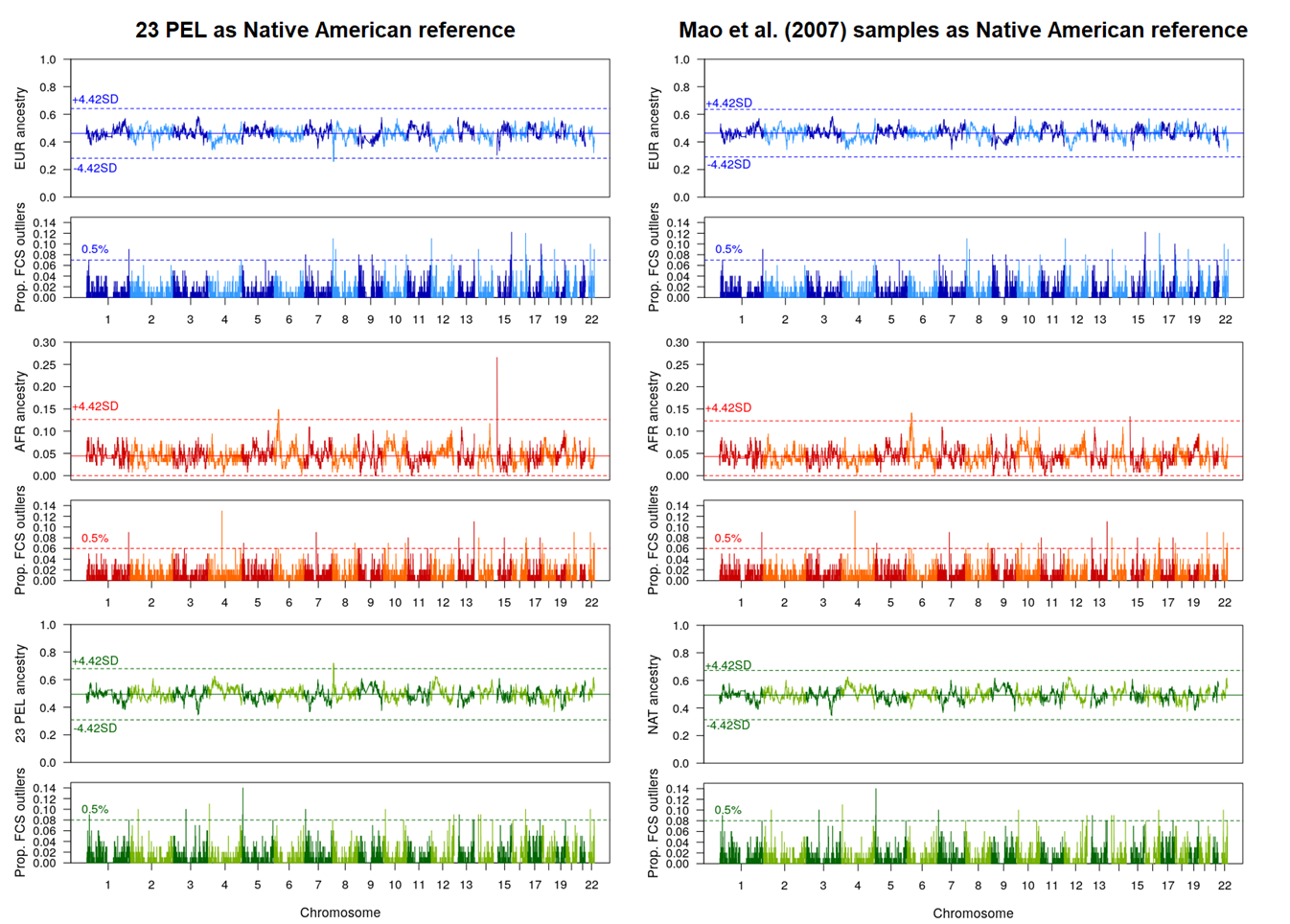


Supplementary Fig. 8. MXL population local ancestry estimation and test of natural selection. We based on NAT ancestry from the 23 PEL individuals and from the 43 individuals from Mao et al., 2007.


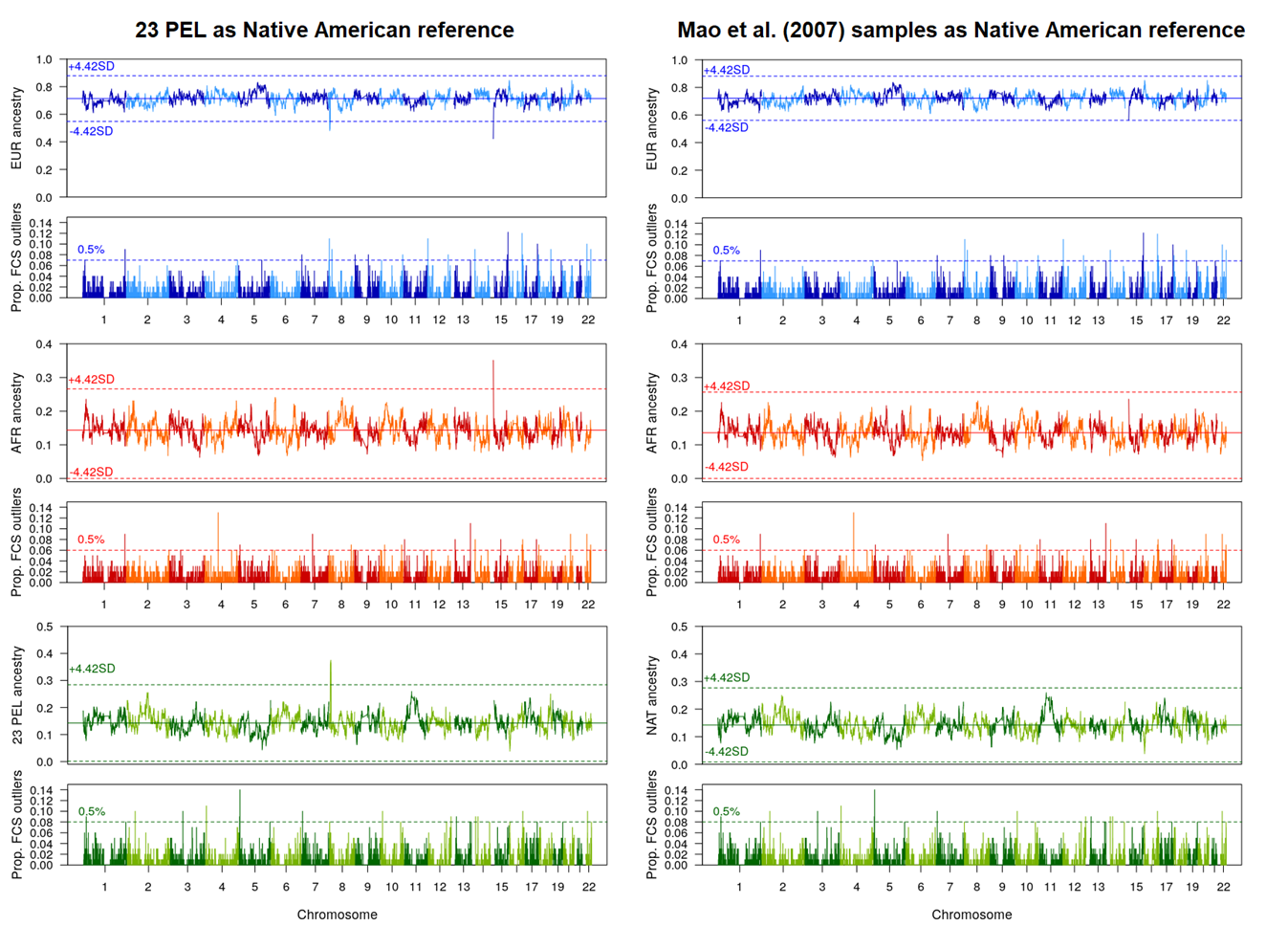


Supplementary Fig. 9. PUR population local ancestry estimation and test of natural selection. We based on NAT ancestry from the 23 PEL individuals and from the 43 individuals from Mao et al., 2007.


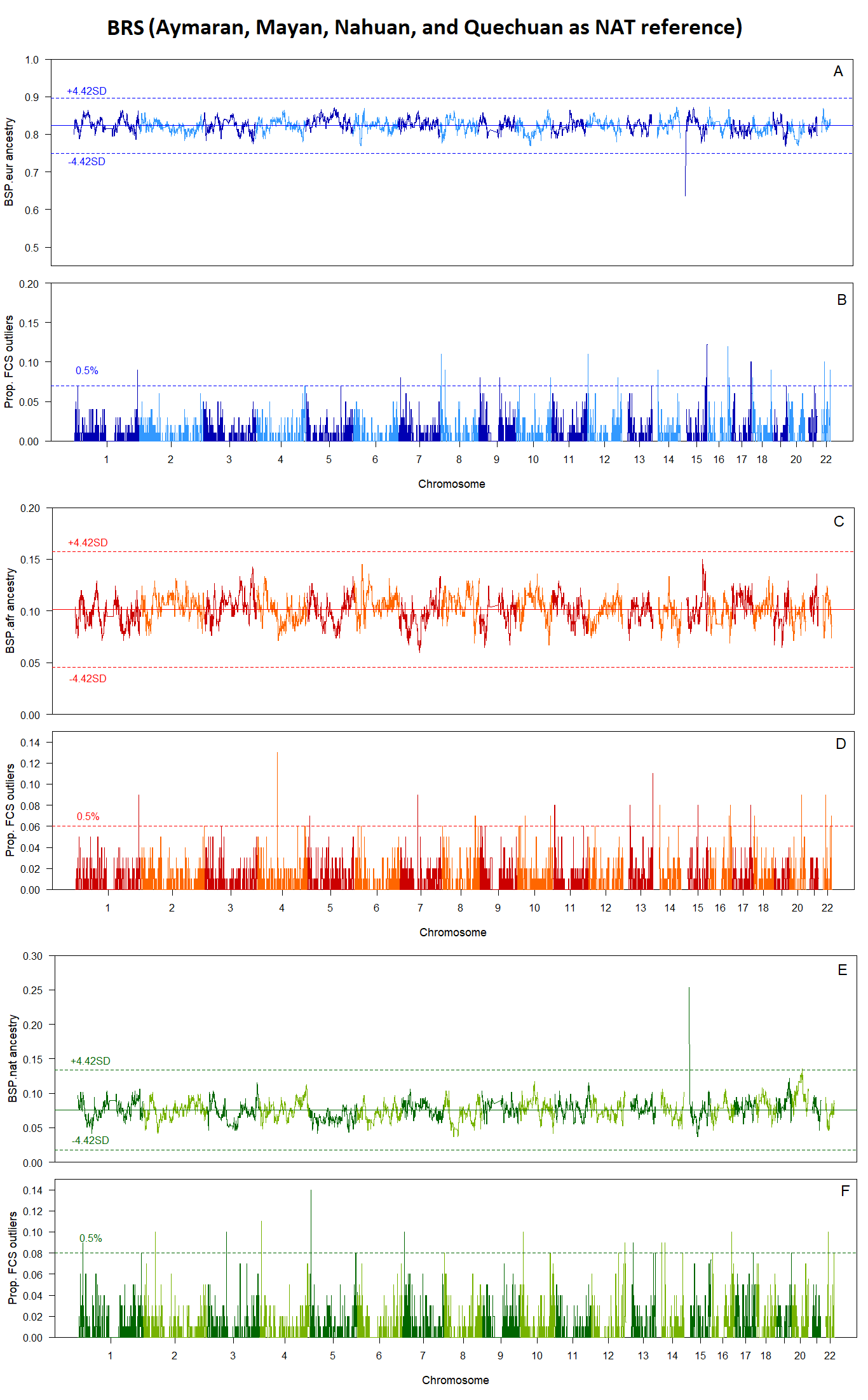


Supplementary Fig. 10. BRS population local ancestry estimation (A, C, E) and test of natural selection (B, D, F), based on NAT ancestry of 43 individuals from Mao et al., 2007.


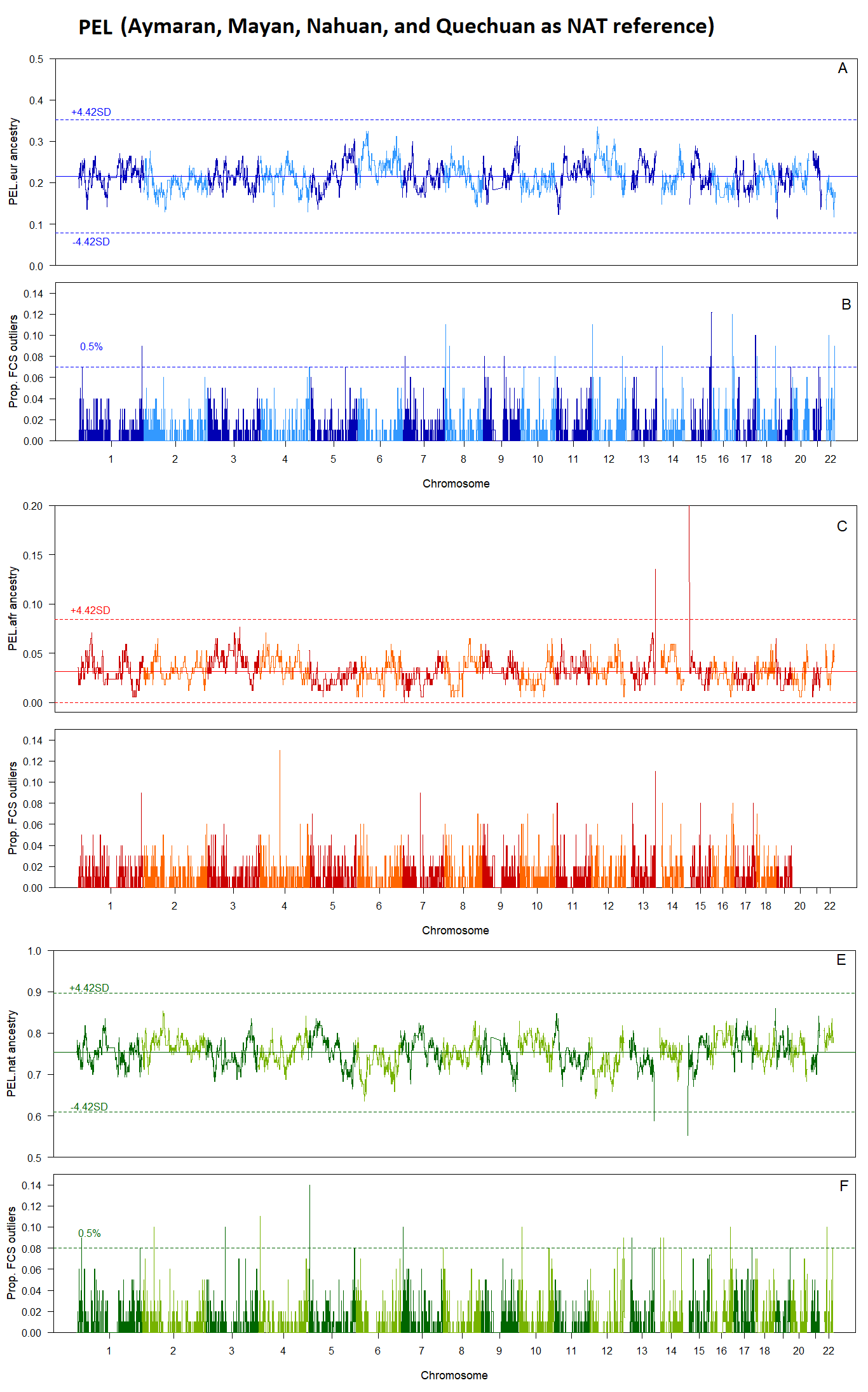


Supplementary Fig. 11. All PEL population local ancestry estimation (A, C, E) and test of natural selection (B, D, F), based on NAT ancestry on 43 individuals from Mao et al., 2007.


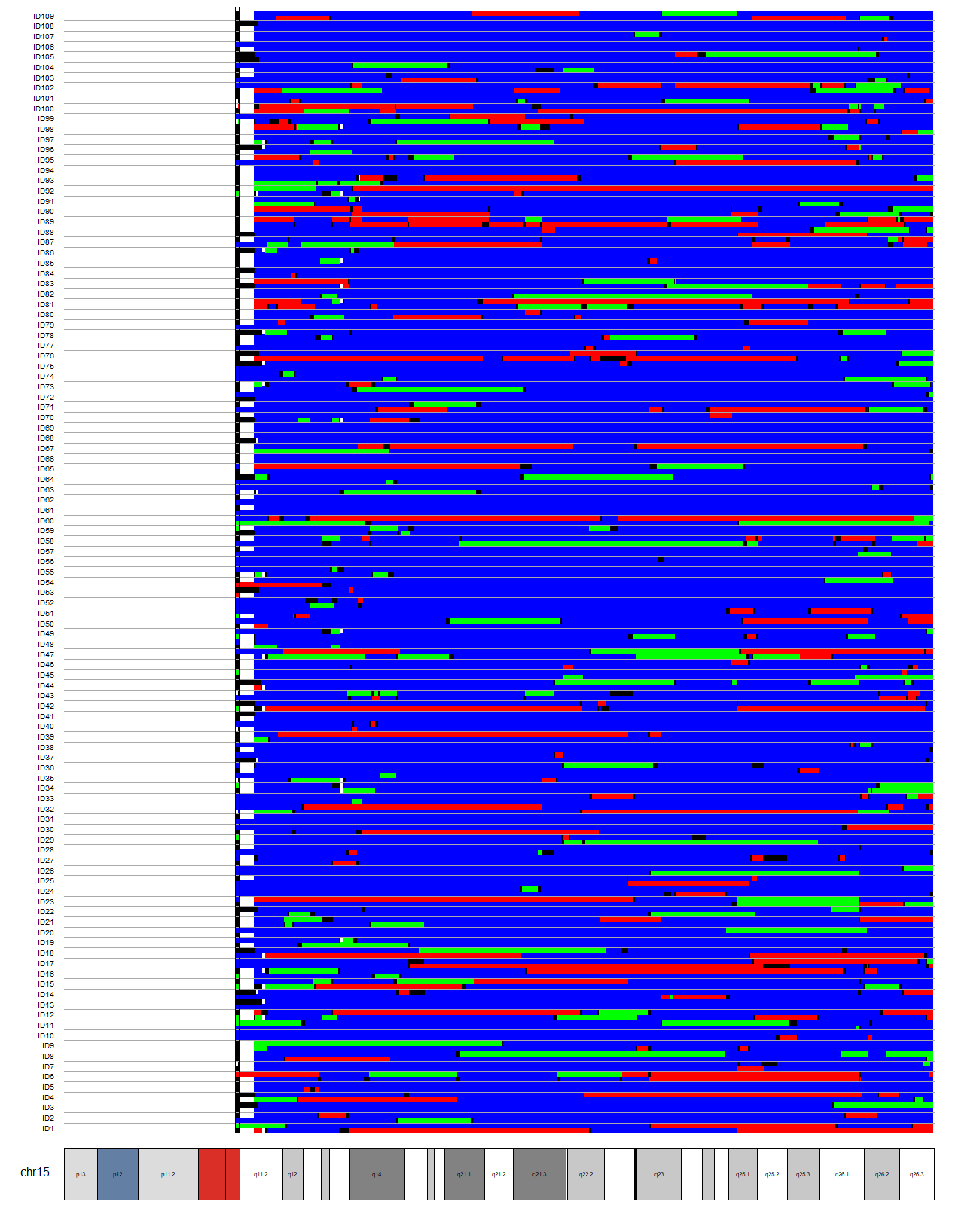


Supplementary Fig. 12. Average ancestry haplotype of chromosome 15 obtained with RFMIX and based on data from 109 BRS individuals. Each colour represents one ancestry component (blue = European; red = African; green = Native-American). Black tracts indicate unknown ancestry (posterior probability < 0.9 according to RFMIX results). We observe a high proportion of unknown blocks on the centromere region (black vertical lines), indicating that unusual local ancestry deviation found here could be due to local ancestry inference bias.


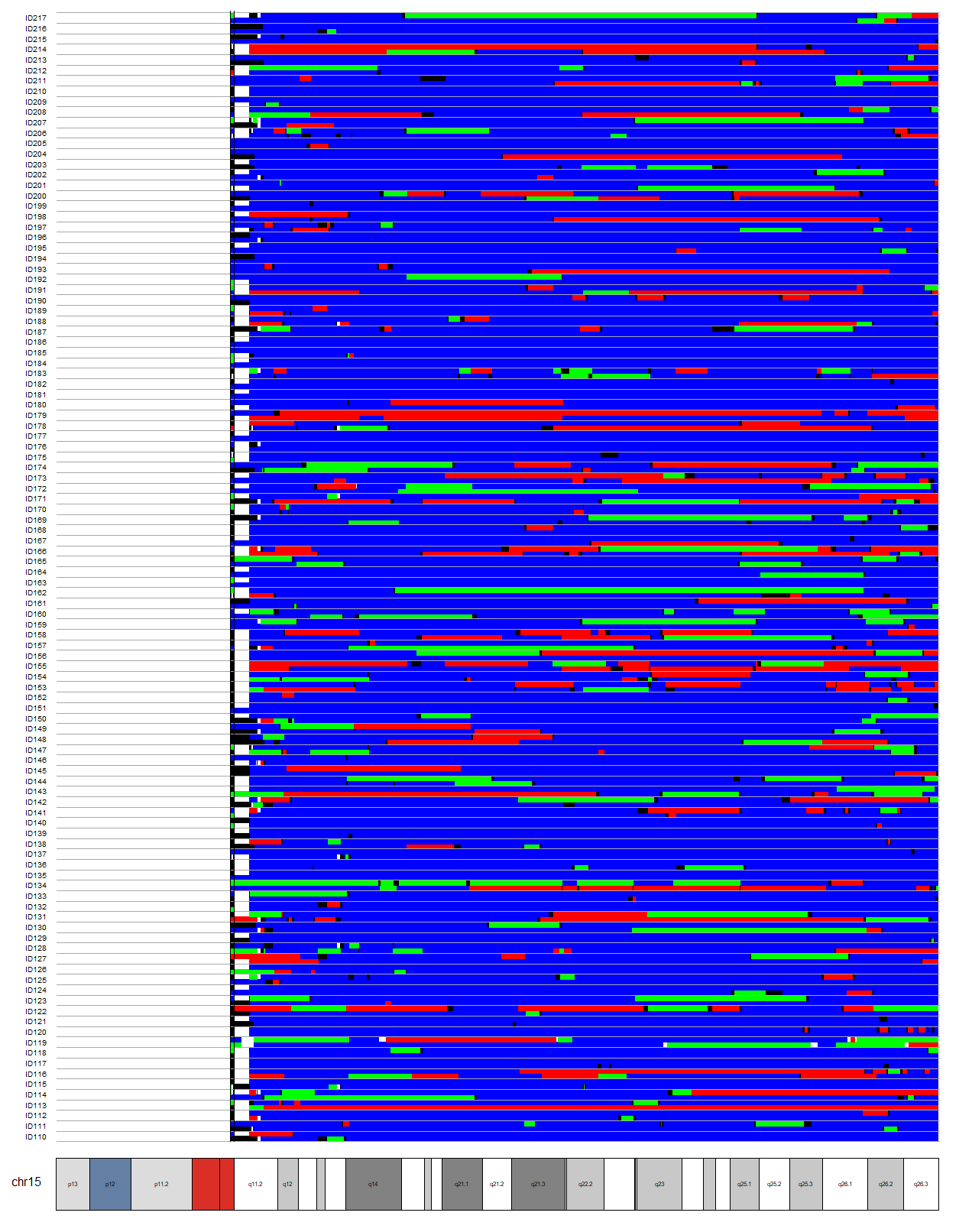


Supplementary Fig. 13. Average ancestry haplotype of chromosome 15 obtained with RFMIX and based on data from the other 108 BRS individuals. Description is the same as in Supplementary Fig. 12.

**
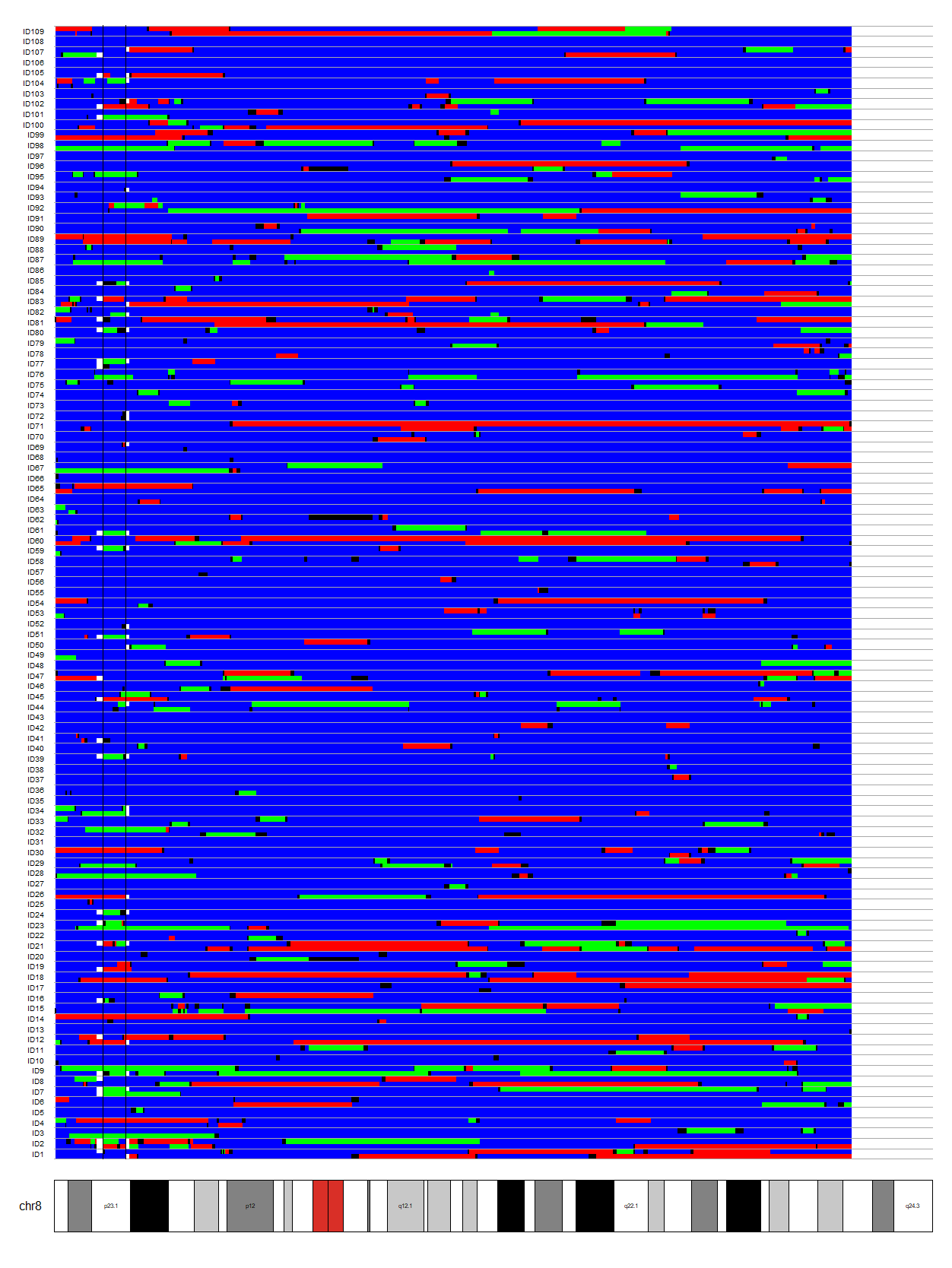
**

Supplementary Fig. 14. Average ancestry haplotype of the locus 8p23.1 (vertical black lines) obtained using RFMIX and based on data from 109 BRS individuals, showing a lower proportion of unknown ancestry tracts. Description is the same as in Supplementary Fig. 12.

**
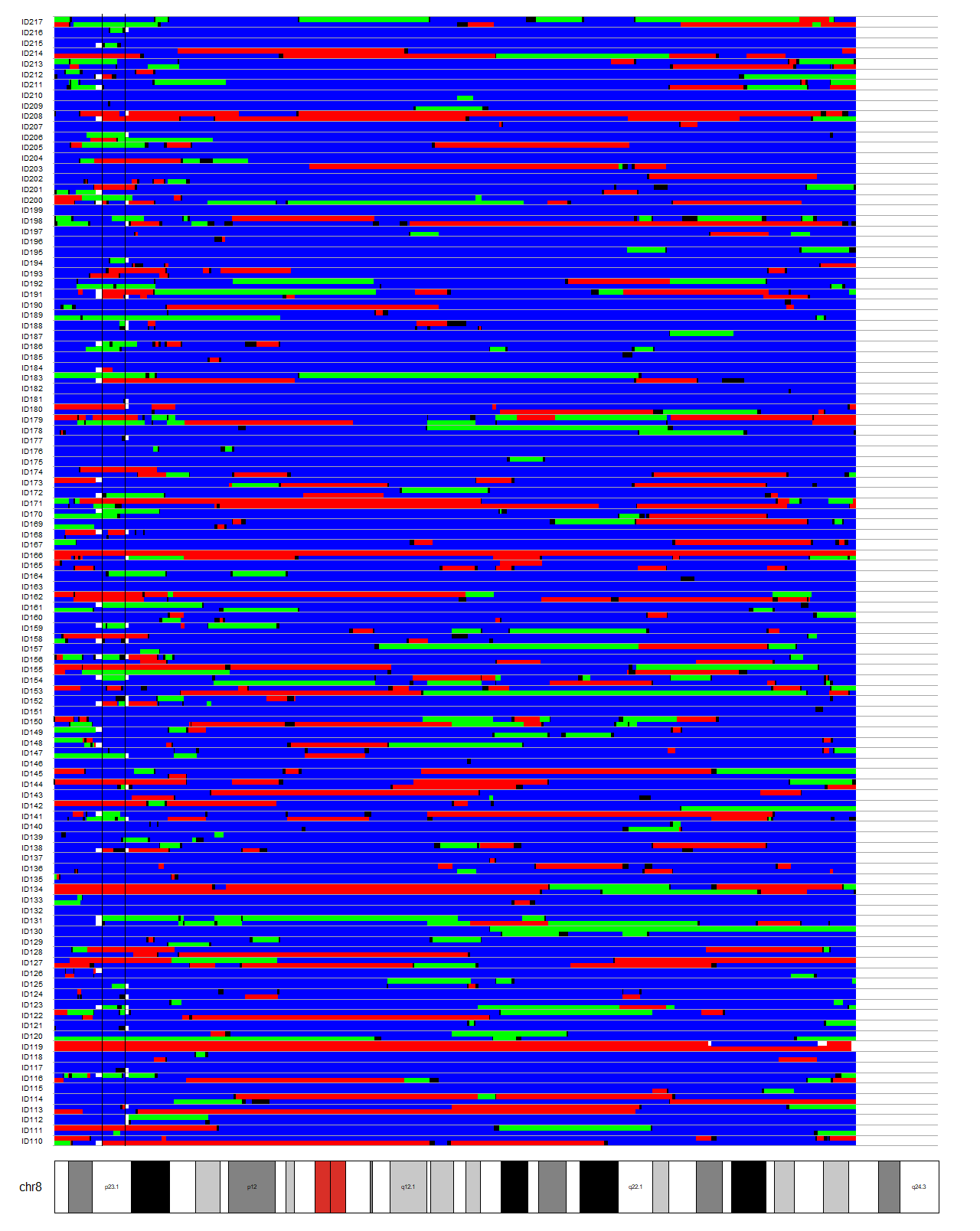
**

Supplementary Fig. 15. Average ancestry haplotype of locus 8p23.1 (vertical black lines) obtained using RFMIX and based on data from the other 108 BRS individuals. Description is the same as in Supplementary Fig. 12.


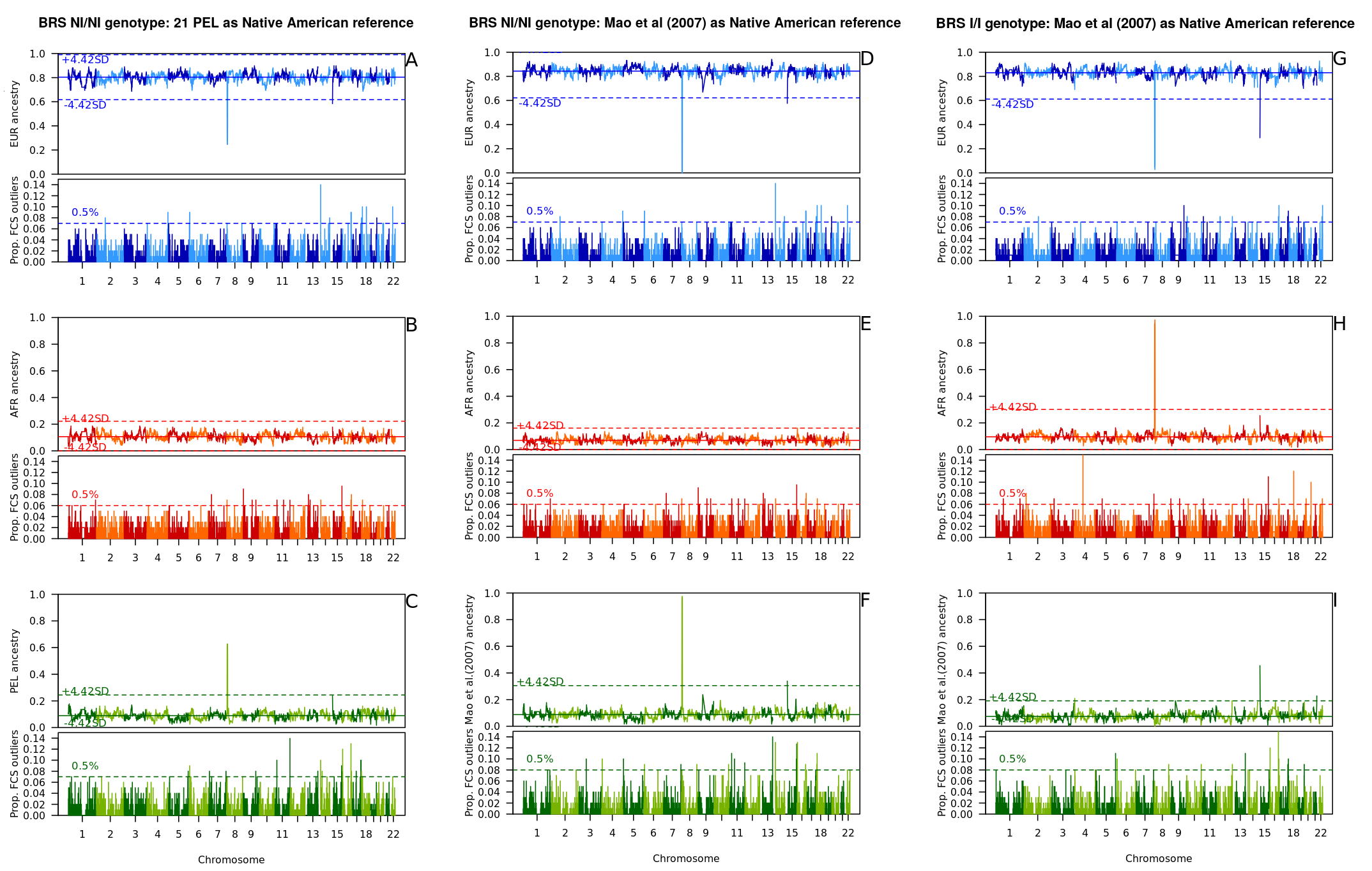


Supplementary Fig. 16. BRS local ancestry inference and positive selection test separated by NI/NI (A–F) and I/I (G–I) genotypes. European (EUR; blue), African (AFR; red), and Native-American (green) references are also based on NI/NI and I/I genotype, separately. We based the Native-American reference on 21 NI/NI Peruvians (A), 17 individuals from Mao et al., 2007 (D, E, F), and five individuals carrying I/I genotypes using Native-Americans from Mao et al., 2007 (G, H, I). We did not observe I/I genotypes in Peruvians to be used as reference.


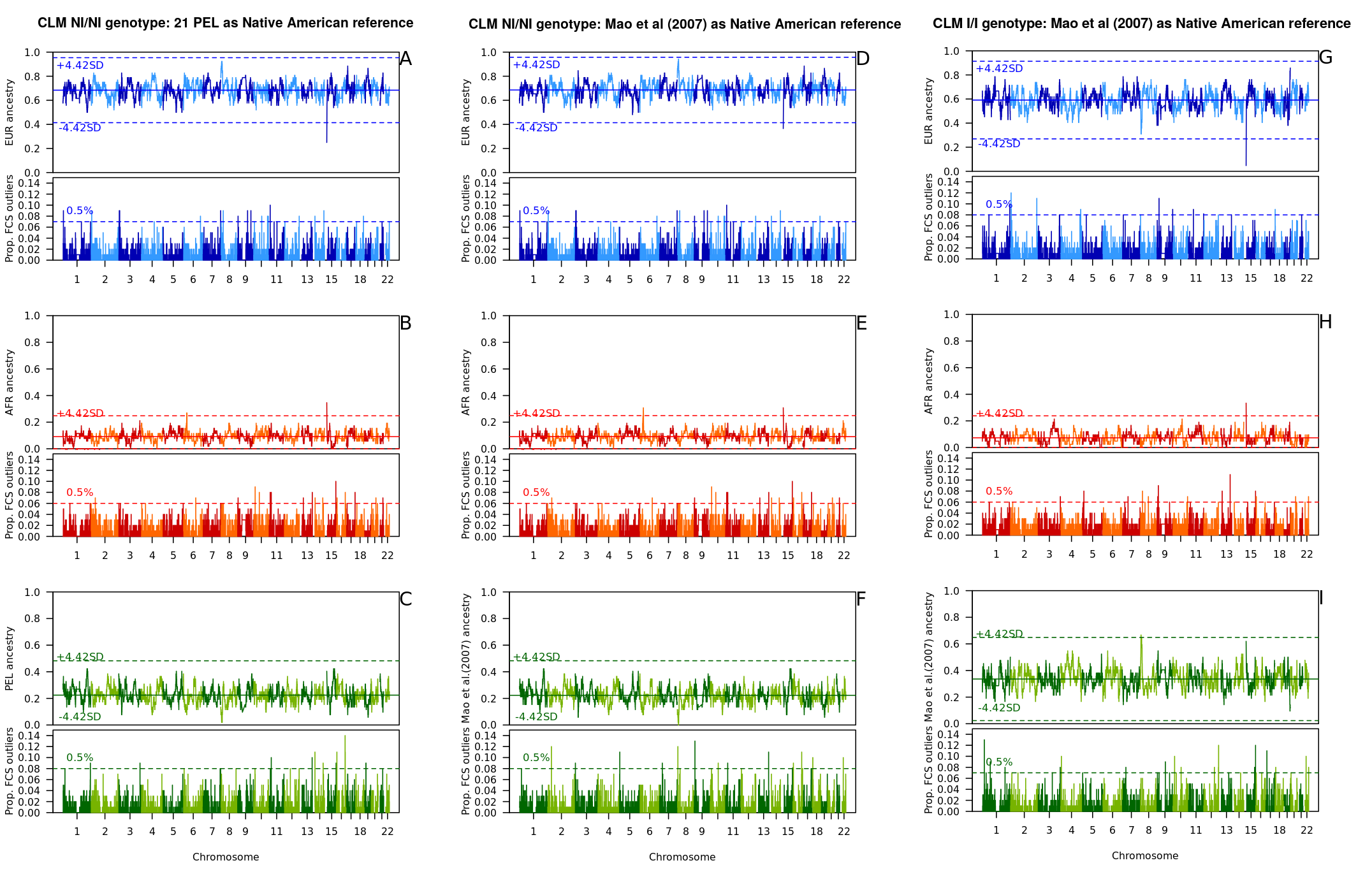


Supplementary Fig. 17. CLM local ancestry inference separated by NI/NI (A–F) and I/I (G–I) genotypes. Figure details are explained in Supplementary Fig. 16.


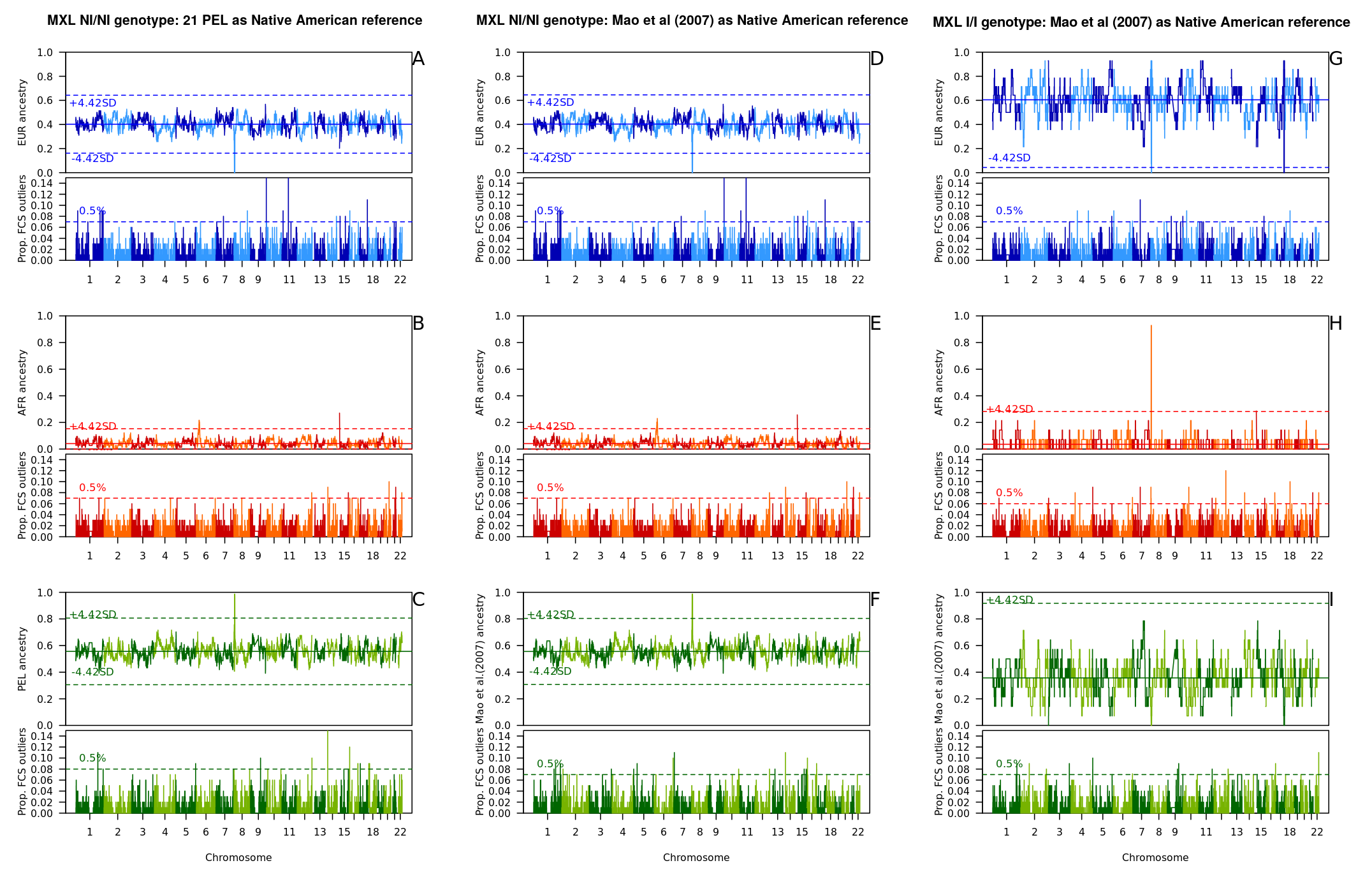


Supplementary Fig. 18. MXL local ancestry inference separated by NI/NI (A–F) and I/I (G–I) genotypes. Figure details are explained in Supplementary Fig. 16.


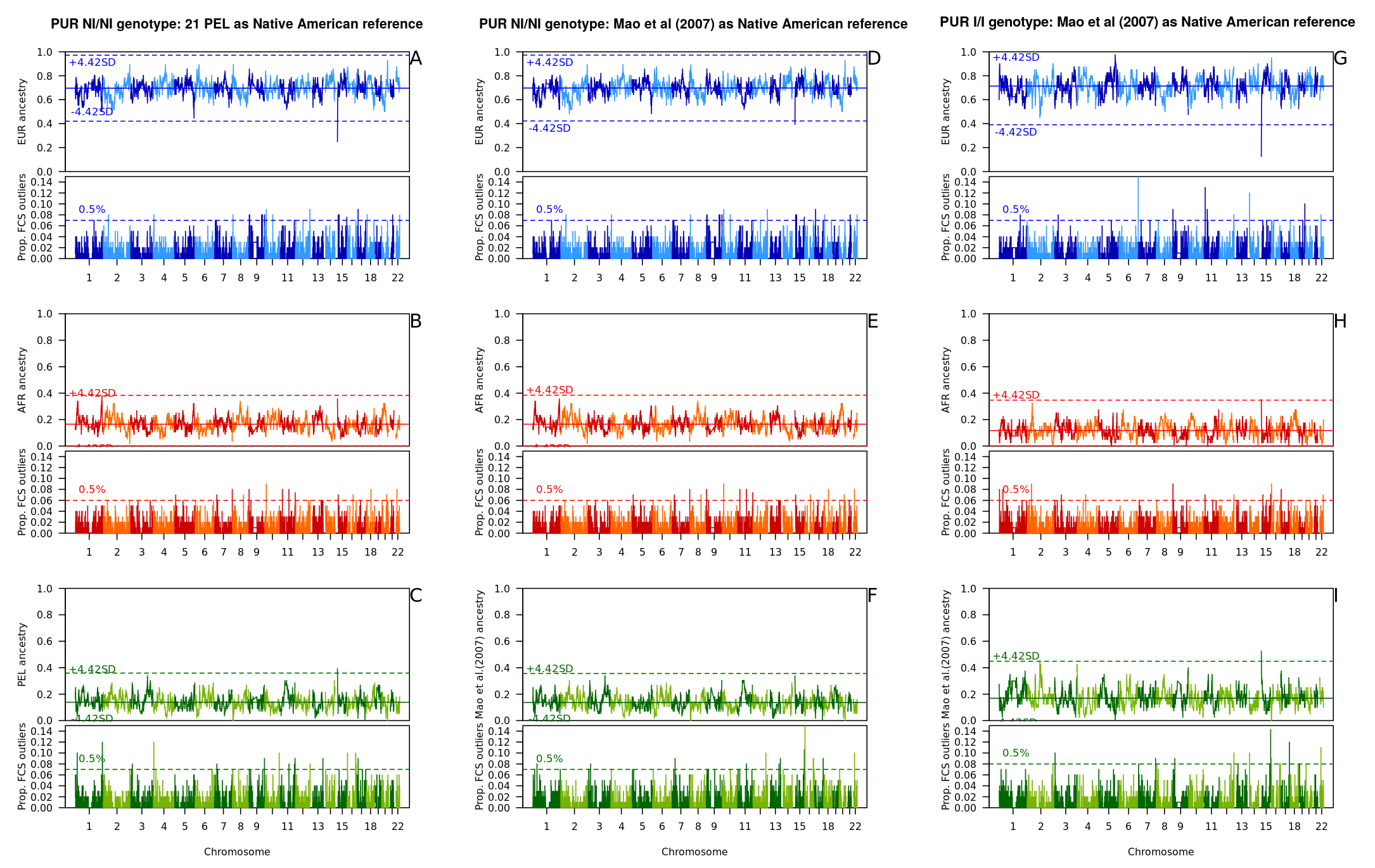


Supplementary Fig. 19. PUR local ancestry inference separated by NI/NI (A–F) and I/I (G–I) genotypes. Figure details are explained in Supplementary Fig. 16.


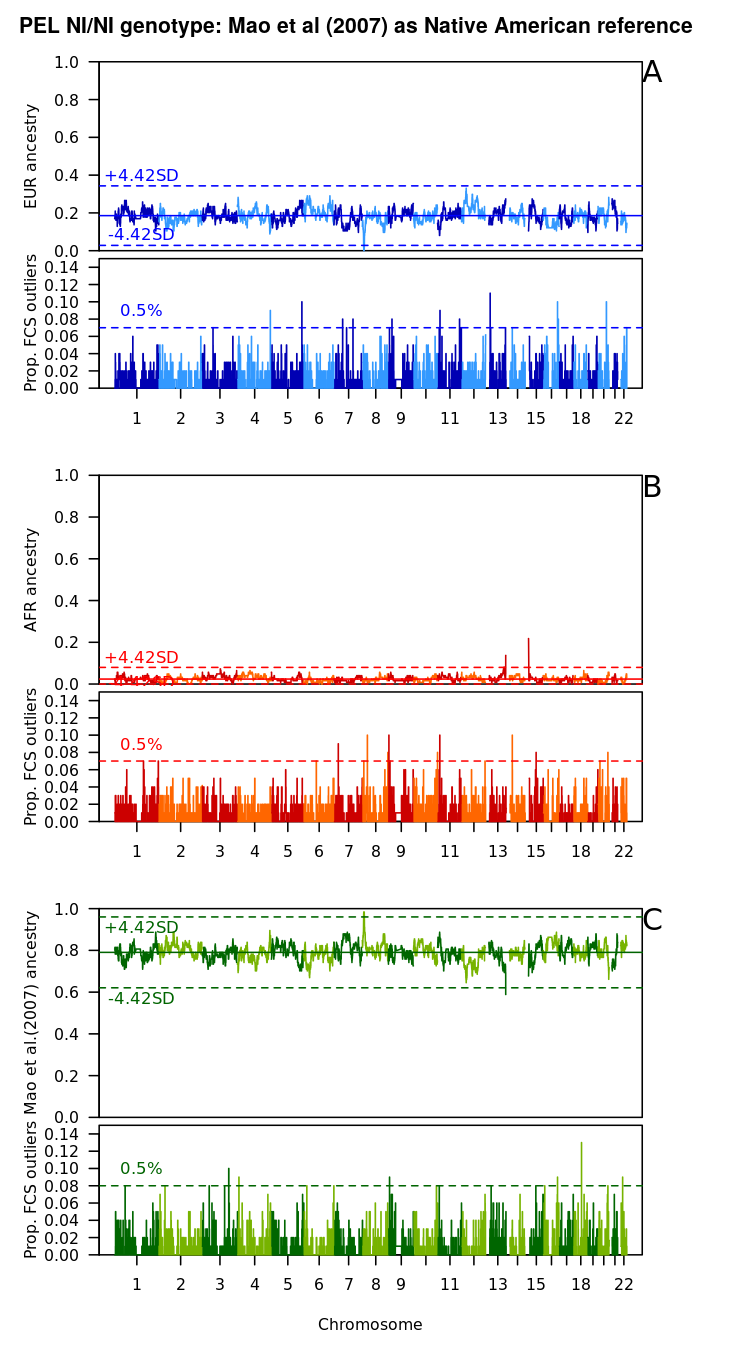


Supplementary Fig. 20. Local ancestry inference from all PEL population separated by NI/NI (A–C) genotypes. European (EUR; blue), African (AFR; red), and Native-American (green) references are also based on NI/NI genotypes. We based the Native-American reference on 17 individuals from Mao et al., 2007.


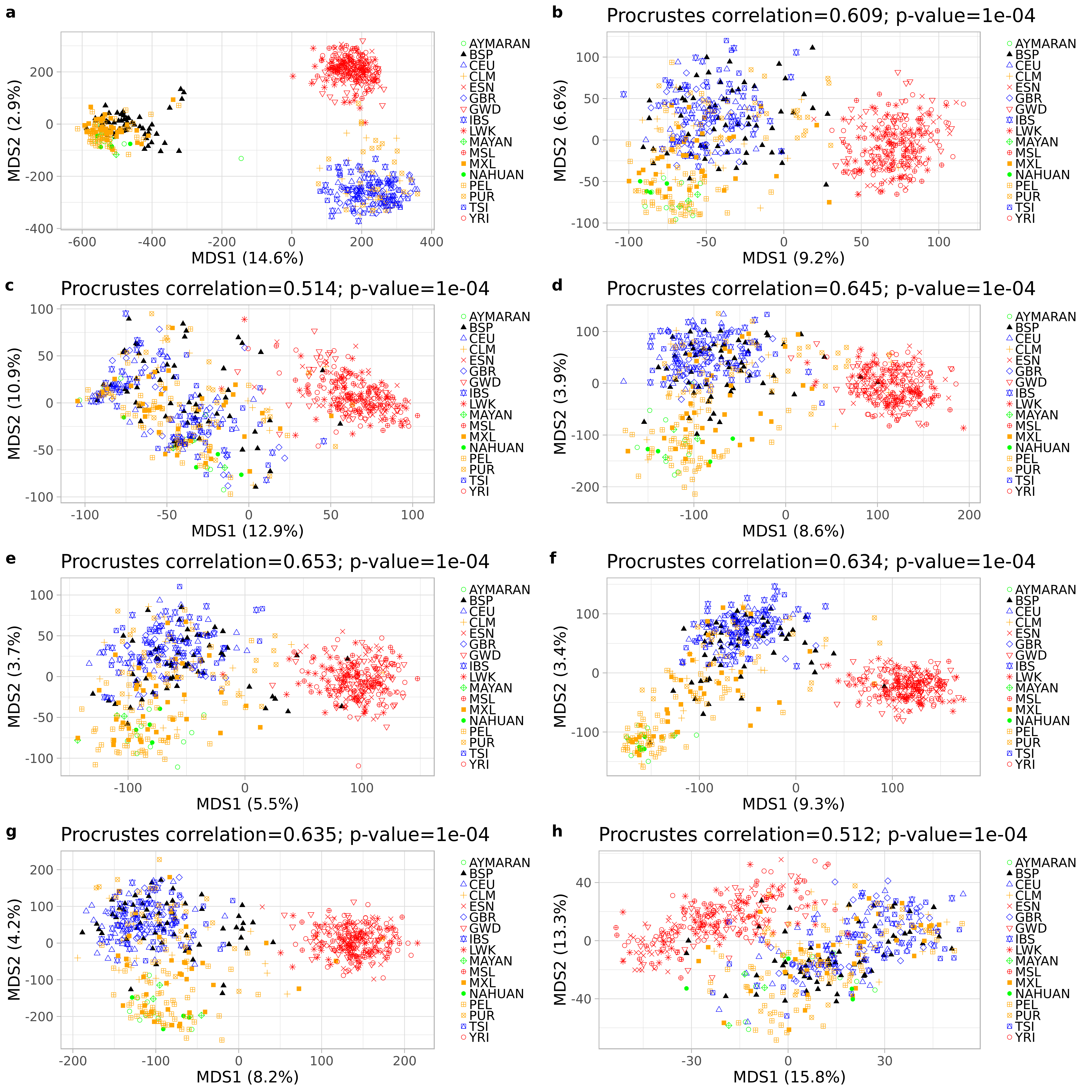


Supplementary Fig. 21. MDS plots based on IBS from NI/NI genotypes, comparing the locus 8p23.1 (1883857 bp) (a), and random loci across the genome of same size as 8p23.1 (b-h). BRS individuals are indicate by black-filled point-up triangles. Each point indicates the MDS1 and MDS2 values based on IBS estimation for each individual, and each colour indicate one continental population (blue = European; red = sub-Saharan African; green = Native-American; orange = admixed American). We compared the similarity between 8p23.1 MDS plot with each random locus MDS plot by Procrustes axis rotation, and the correlation values and the respective p-value for each random locus is showed (b-h).


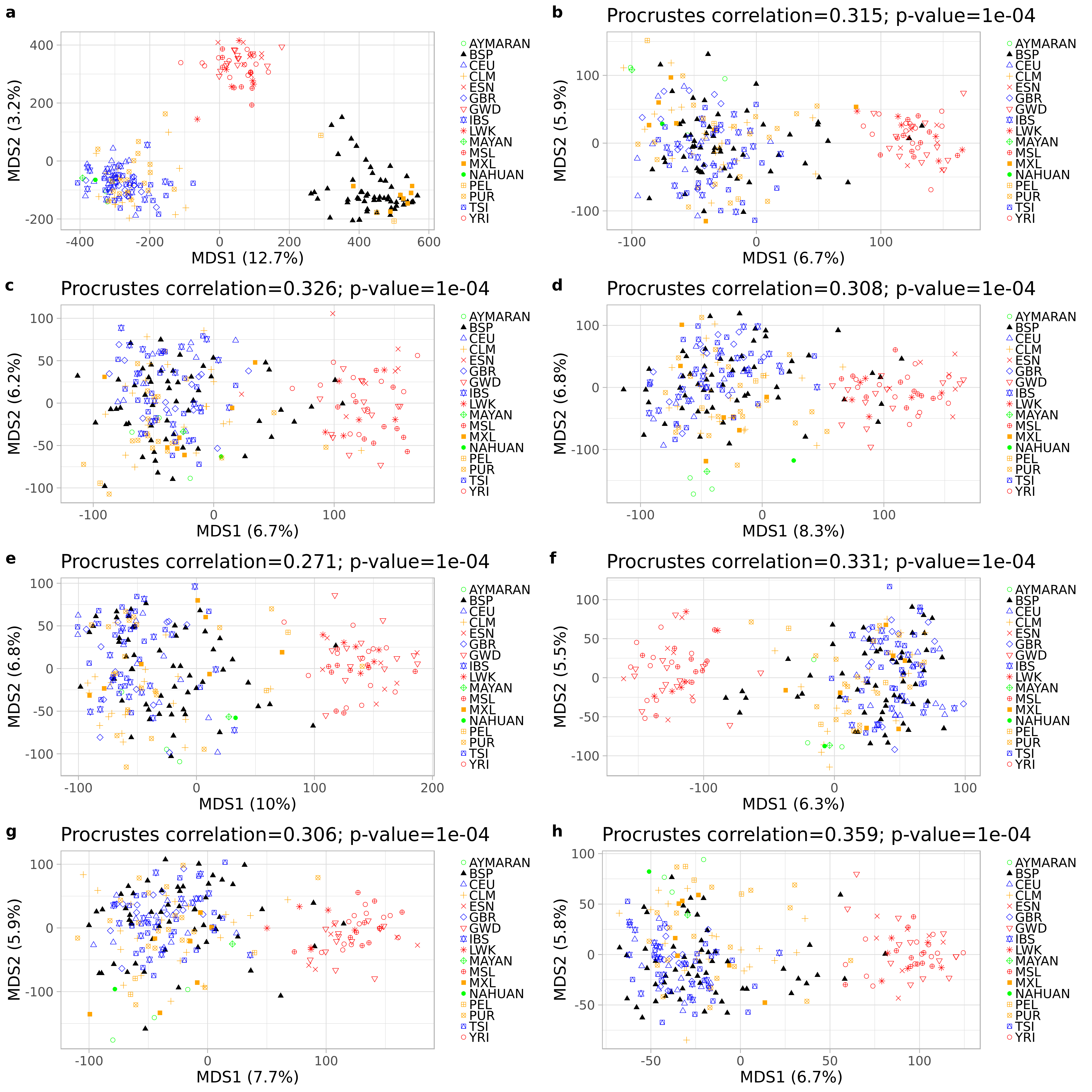


Supplementary Fig. 22. MDS plots based on IBS from I/I genotypes, comparing the locus 8p23.1 (1883857 bp) (a), and random loci across the genome of same size as 8p23.1 (b-h). BRS individuals are indicate by black-filled point-up triangles. Each point indicates the MDS1 and MDS2 values based on IBS estimation for each individual, and each colour indicate one continental population (blue = European; red = sub-Saharan African; green = Native-American; orange = admixed American). We compared the similarity between 8p23.1 MDS plot with each random locus MDS plot by Procrustes axis rotation, and the correlation values and the respective p-value for each random locus is showed (b-h).


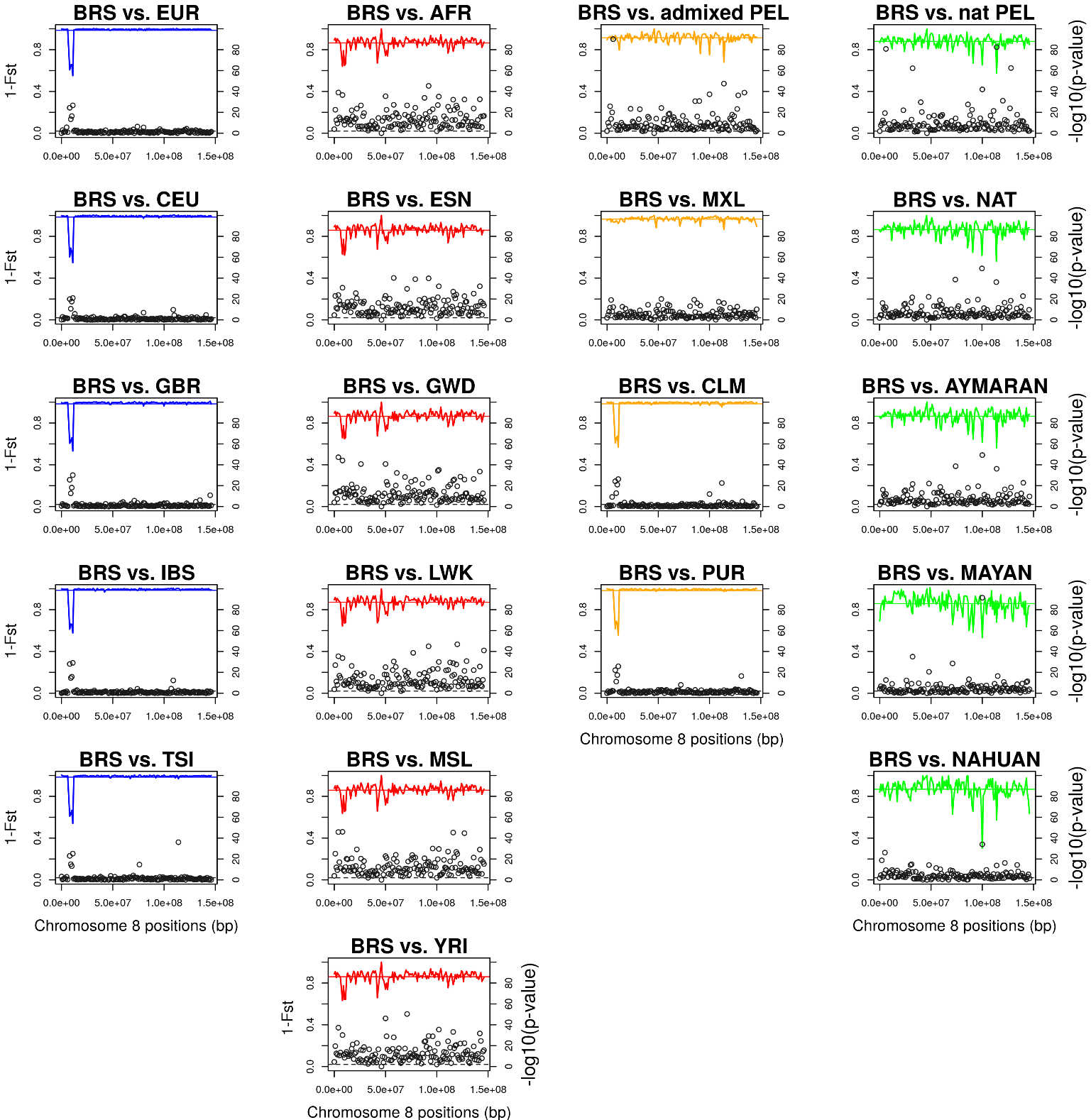


Supplementary Fig. 23. Estimates of 1-Fst for NI/NI genotypes based on 1 Mb windows among chromosome 8 comparing BRS sample with European (blue), African (red), admixed American (orange), and native-American (green) from Mao et al., 2007. The lower the 1-Fst value, the more different the two populations. Solid lines indicate the mean of 1-fst values, and dashed lines indicates 4 SDs threshold. Each grey point indicates the –log10(p-values) from Fst for each window, dashed grey line indicates p-value threshold for Fst (–log10(0.01)), and points above this line indicates significant Fst estimates. Quechuan sample was not included due to lack of NI/NI genotypes.

**
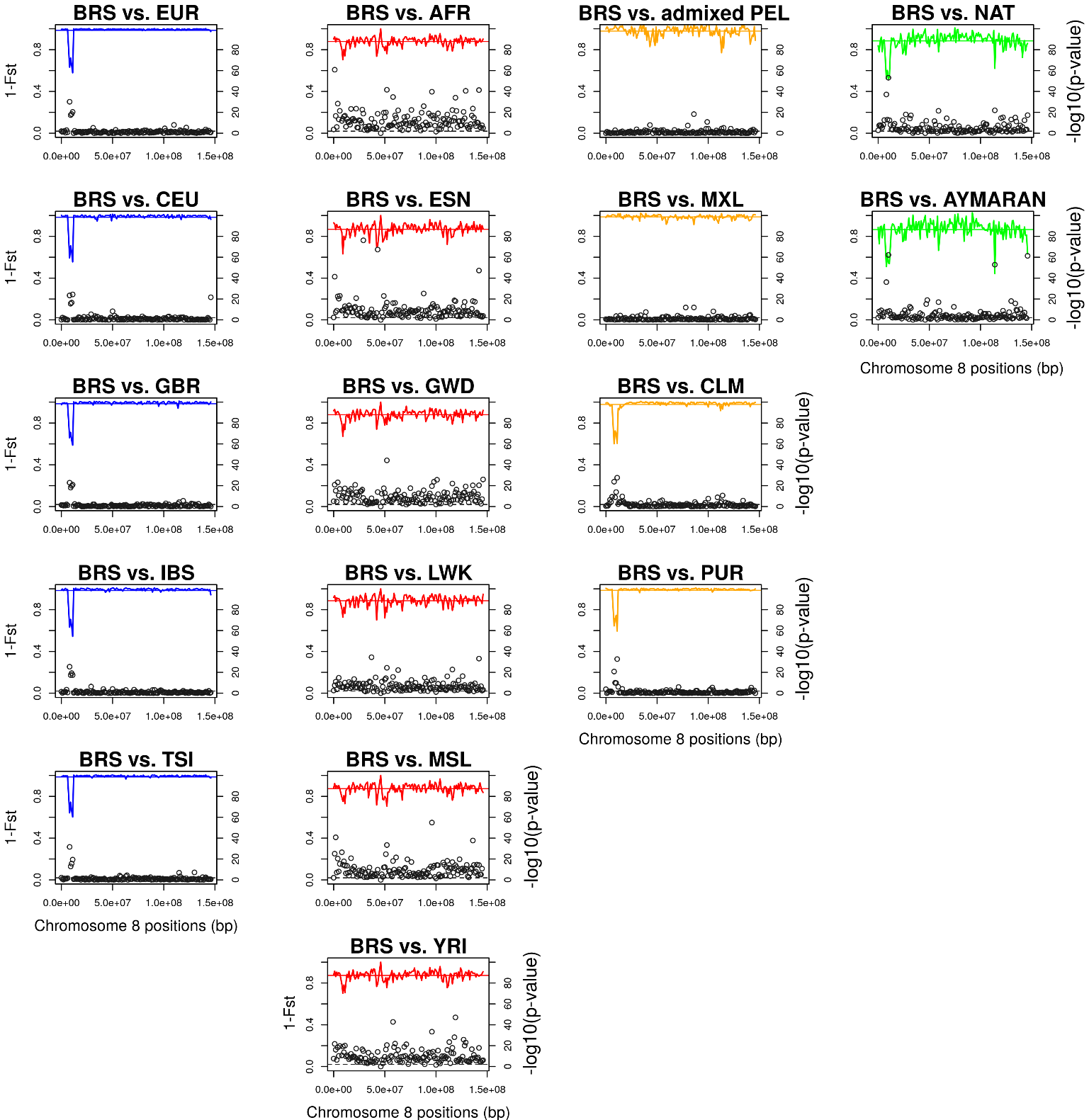
**

Supplementary Fig. 24. Estimates of 1-Fst for I/I genotypes based on 1 Mb windows among chromosome 8 comparing BRS sample with European (blue), African (red), admixed American (orange), and native-American (green) from Mao et al., 2007. The lower the 1-Fst value, the more different the two populations. Solid lines indicate the mean of 1-fst values, and dashed lines indicates 4 SDs threshold. Each grey point indicates the –log10(p-values) from Fst for each window, dashed grey line indicates p-value threshold for Fst (–log10(0.01)), and points above this line indicates significant Fst estimates. No-admixed Peruvian, Quechuan, Mayan, and Nahuan samples were not included due to lack of enough I/I genotype for Fst estimation.


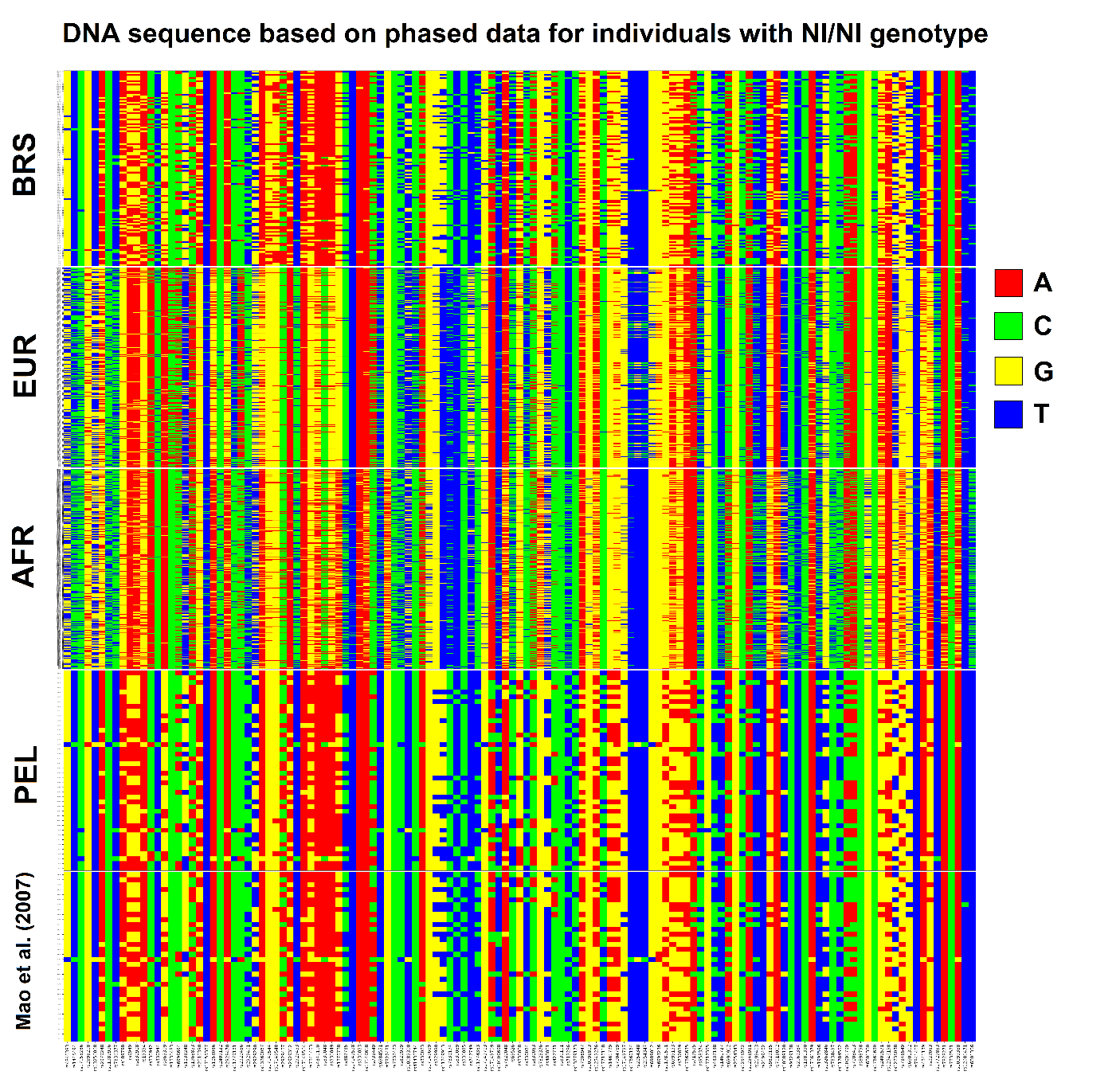


Supplementary Fig. 25. DNA sequence along the 8962666bp-9265464bp region which presented positive selection signals and based on phased data for individuals with NI/NI genotype. Each colour indicates one allele (A=red; C=green; G=yellow; T=blue). Each vertical bar represents the alleles for each of the 131 SNPs on the region (x-axis). Each horizontal bar represents the sequence of alleles for each individual from different continental population (y-axis).


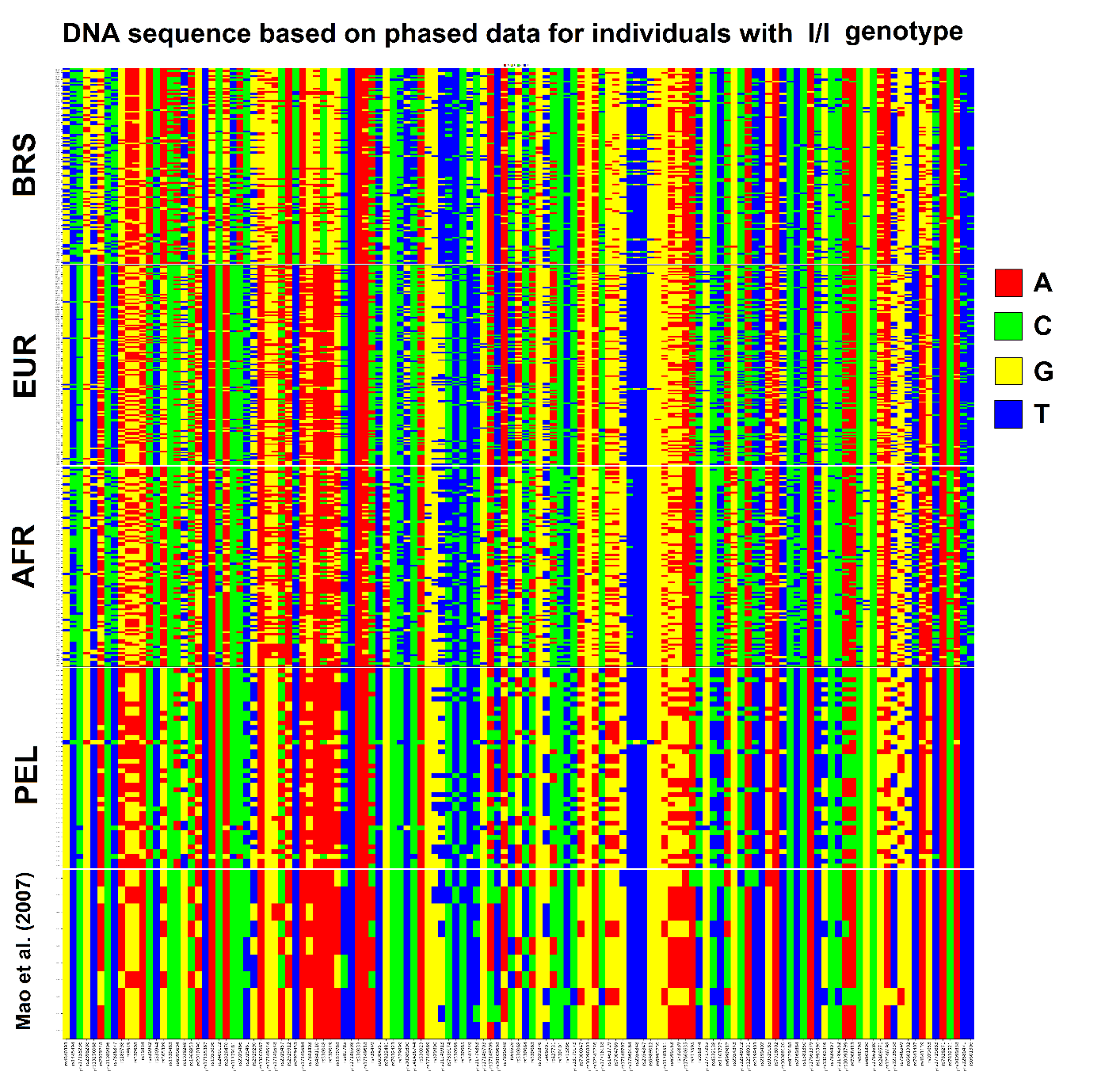


Supplementary Fig. 26. DNA sequence along the 8962666bp-9265464bp region which presented positive selection signals and based on phased data for individuals with I/I genotype. Each colour indicates one allele (A=red; C=green; G=yellow; T=blue). Each vertical bar represents the alleles for each of the 131 SNPs on the region (x-axis). Each horizontal bar represents the sequence of alleles for each individual from different continental population (y-axis).

**
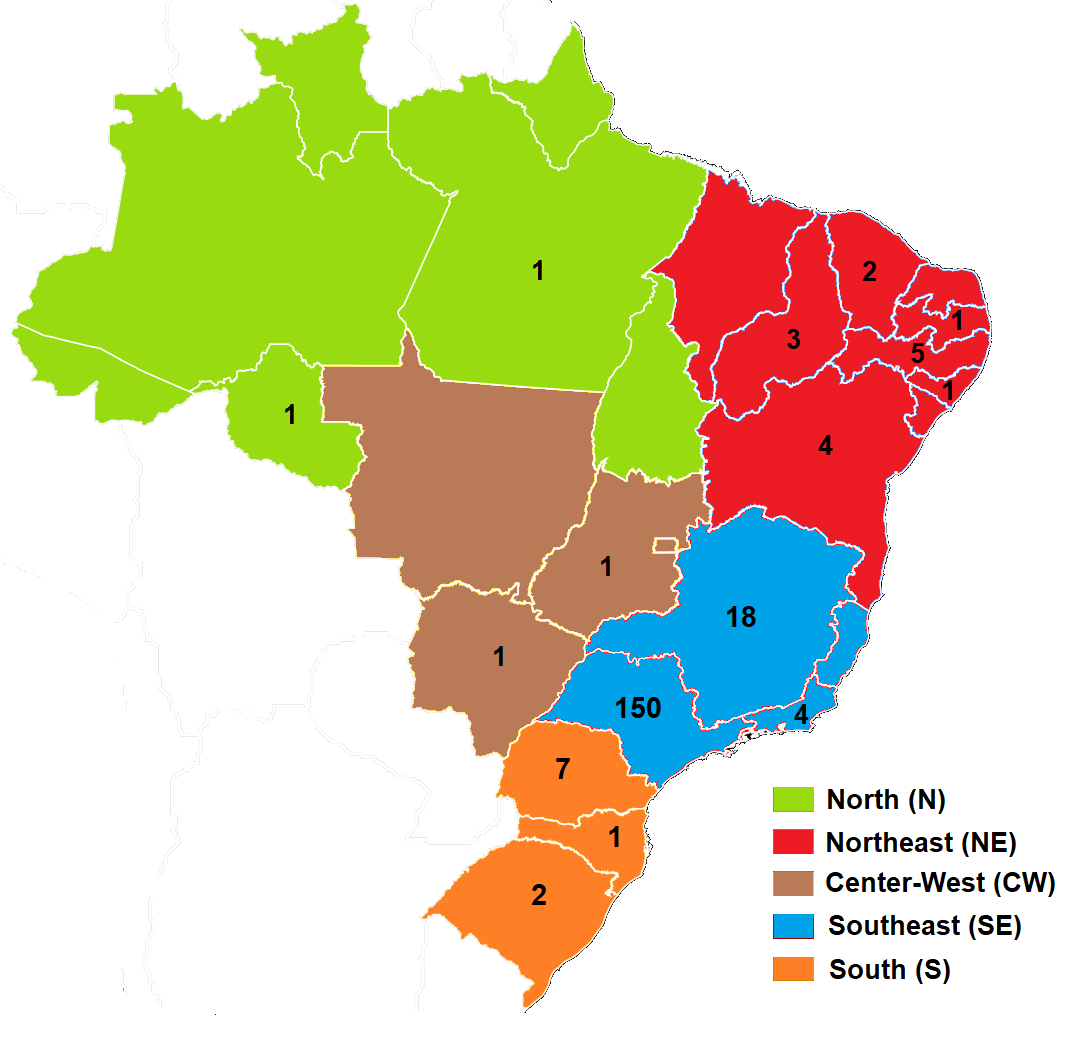
**

Supplementary Fig. 27. Map of Brazil indicating the geographic distribution of the 202 BRS samples who have known place of birth. Each colour indicates one Brazilian geographic region. The black numbers indicate the number of individuals born in each Brazilian state.


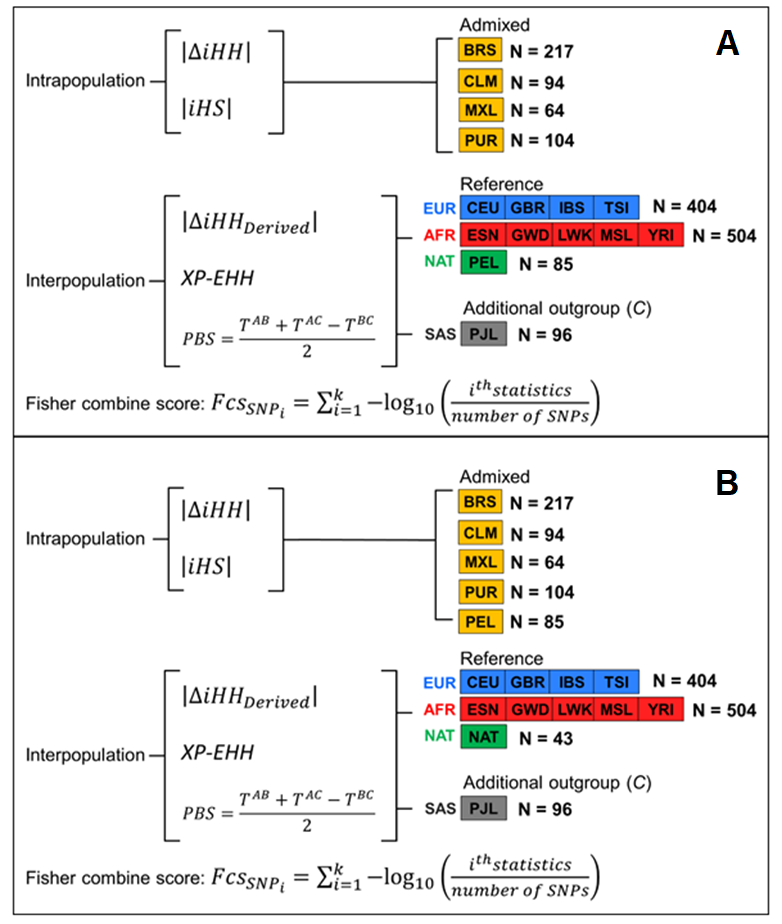


Supplementary Fig. 28. Selection tests study design. Reference panel selection for local ancestry inference included Europeans (blue squares), Africans (red squares), and the Peruvian individuals as Native-American reference (green square) in the first approach (A); and 43 Native-Americans (2,3) in the second approach (B), which allowed us to include the Peruvians in the analysis. For PBS, we include the PJL as an outgroup population. We removed from the analysis the five BRS individuals with Japanese ancestry t, shown in Figure 1.

Supplementary Table 1. Mean, standard deviation (SD), and 95% confidence intervals (CI) of ancestry proportions in the BRS sample for K = 5 and K = 10 ADMIXTURE values.

| **K** | **Ancestry component** | **Mean** | **SD** | **95% CI** |
| --- | --- | --- | --- | --- |
| **5** | European | 0.769 | 0.157 | 0.748–0.790 |
|  | Sub-Saharan African | 0.138 | 0.116 | 0.122–0.153 |
|  | Native-American | 0.070 | 0.056 | 0.062–0.077 |
|  | East Asian | 0.003 | 0.009 | 0.002–0.004 |
|  | South Asian | 0.020 | 0.069 | 0.011–0.029 |
| **10** | South European (IBS, TSI) | 0.689 | 0.144 | 0.670–0.709 |
|  | West African (YRI, ESN) | 0.077 | 0.092 | 0.065–0.090 |
|  | Native-American | 0.076 | 0.058 | 0.068–0.083 |
|  | East African (LWK) | 0.065 | 0.068 | 0.056–0.074 |
|  | North European (FIN) | 0.059 | 0.063 | 0.051–0.068 |
|  | East Asian (JPT) | 0.017 | 0.071 | 0.007–0.026 |
|  | West African (GWD) | 0.009 | 0.018 | 0.006–0.011 |
|  | East Asian (CDX) | 0.003 | 0.005 | 0.003–0.004 |
|  | South Asian | 0.003 | 0.006 | 0.002–0.004 |
|  | South Asian (GIH) | 0.002 | 0.007 | 0.001–0.003 |

Supplementary Table 2. AMOVA results based on genetic distance among BRS sample, classified according State of birth, including Monte Carlo test p-values. Values were estimated based on 10,000 (10k) random SNPs and separately by chromosome.

| **Chrosomosome** | **Variation component (%)** | | **Total φ-statistics** | **p-value** |
| --- | --- | --- | --- | --- |
|  | **Within-group** | **Among-group** |  |  |
| 10k random SNPs | 99.98 | 0.02 | 0.0002 | 0.2327 |
| Chr. 1 | 99.99 | 0.01 | 0.00005 | 0.3956 |
| Chr. 2 | 100.00 | 0.00 | 0.0001 | 0.5305 |
| Chr. 3 | 99.97 | 0.03 | 0.0003 | 0.1958 |
| Chr. 4 | 99.98 | 0.02 | 0.0002 | 0.2647 |
| Chr. 5 | 100.00 | 0.00 | 0.00001 | 0.4256 |
| Chr. 6 | 100.00 | 0.00 | 0.00003 | 0.4036 |
| Chr. 7 | 100.00 | 0.00 | 0.0003 | 0.7632 |
| Chr. 8 | 99.95 | 0.05 | 0.0005 | 0.1389 |
| Chr. 9 | 100.00 | 0.00 | 0.0001 | 0.5504 |
| Chr. 10 | 100.00 | 0.00 | 0.00003 | 0.4266 |
| Chr. 11 | 100.00 | 0.00 | 0.00005 | 0.3986 |
| Chr. 12 | 100.00 | 0.00 | 0.00003 | 0.3886 |
| Chr. 13 | 99.96 | 0.04 | 0.0004 | 0.1808 |
| Chr. 14 | 99.99 | 0.01 | 0.0001 | 0.3337 |
| Chr. 15 | 99.98 | 0.02 | 0.0002 | 0.2697 |
| Chr. 16 | 100.00 | 0.00 | 0.00009 | 0.5514 |
| Chr. 17 | 99.98 | 0.02 | 0.0002 | 0.2667 |
| Chr. 18 | 99.98 | 0.02 | 0.0002 | 0.3427 |
| Chr. 19 | 99.97 | 0.03 | 0.0003 | 0.2138 |
| Chr. 20 | 100.00 | 0.00 | 0.0003 | 0.6993 |
| Chr. 21 | 99.95 | 0.05 | 0.0004 | 0.1618 |
| Chr. 22 | 100.00 | 0.00 | 0.0003 | 0.7192 |

Supplementary Table 3. Distribution of chromosome 8p23.1 inversion genotypes and ancestry tracts among Brazilian individuals (BRS) and other admixed American populations.

| **Pop** | **Genotypes** | **Chr8p23.1 ancestry tract** | | | | | | | **Total** |
| --- | --- | --- | --- | --- | --- | --- | --- | --- | --- |
|  |  | **AFR** | **EUR** | **NAT** | **AFR-EUR** | **AFR-NAT** | **EUR-NAT** | **AFR-EUR-NAT** |  |
| **BRS** | **I/I** | 1 | 48 | 0 | 6 | 0 | 0 | 0 | 55 |
|  | **NI/I** | 3 | 45 | 0 | 25 | 2 | 19 | 9 | 103 |
|  | **NI/NI** | 0 | 21 | 2 | 2 | 2 | 31 | 1 | 59 |
|  | **Total** | 4 | 114 | 2 | 33 | 4 | 50 | 10 | 217 |
| **CLM** | **I/I** | 0 | 3 | 7 | 0 | 0 | 10 | 1 | 21 |
|  | **NI/I** | 0 | 17 | 0 | 3 | 0 | 22 | 5 | 47 |
|  | **NI/NI** | 0 | 21 | 0 | 5 | 0 | 0 | 0 | 26 |
|  | **Total** | 0 | 41 | 7 | 8 | 0 | 32 | 6 | 94 |
| **MXL** | **I/I** | 0 | 7 | 0 | 0 | 0 | 0 | 0 | 7 |
|  | **NI/I** | 0 | 8 | 0 | 0 | 1 | 9 | 2 | 20 |
|  | **NI/NI** | 0 | 1 | 17 | 0 | 1 | 18 | 0 | 37 |
|  | **Total** | 0 | 16 | 17 | 0 | 2 | 27 | 2 | 64 |
| **PUR** | **I/I** | 0 | 10 | 0 | 2 | 0 | 8 | 0 | 20 |
|  | **NI/I** | 2 | 25 | 0 | 12 | 1 | 12 | 4 | 56 |
|  | **NI/NI** | 0 | 15 | 0 | 13 | 0 | 0 | 0 | 28 |
|  | **Total** | 2 | 50 | 0 | 27 | 1 | 20 | 4 | 104 |
| **nat PEL*** | **I/I** | 0 | 0 | 0 | 0 | 0 | 0 | 0 | 0 |
|  | **NI/I** | 0 | 0 | 0 | 0 | 1 | 1 | 0 | 2 |
|  | **NI/NI** | 0 | 0 | 18 | 0 | 1 | 2 | 0 | 21 |
|  | **Total** | 0 | 0 | 18 | 0 | 2 | 3 | 0 | 23 |

*Ancestry tracts extract from 23 Peruvians using the Native-American from Mao et al., 2007 as reference. I: inverted, and NI: non-inverted haplotypes.

Supplementary Table 4. Fst values for the NI/NI genotypes from the chromosome 8p23.1 region (1298 SNPs). The darker the colour, the higher the Fst (i.e. more genetic differentiation). Ayamarans (AYM), Mayans (MAY), and Nahuans (NAH) were included in the analysis since there were enough individuals available to compute Fst values.

| **Pop** | **BRS** | **AYM** | **MAY** | **NAH** | **PEL** | **MXL** | **CLM** | **PUR** | **TSI** | **IBS** | **CEU** | **GBR** | **FIN** | **ESN** | **GWD** | **LWK** | **MSL** | **YRI** |
| --- | --- | --- | --- | --- | --- | --- | --- | --- | --- | --- | --- | --- | --- | --- | --- | --- | --- | --- |
| **BRS** | 0.000 |  |  |  |  |  |  |  |  |  |  |  |  |  |  |  |  |  |
| **AYM** | 0.100 | 0.000 |  |  |  |  |  |  |  |  |  |  |  |  |  |  |  |  |
| **MAY** | 0.098 | 0.000 | 0.000 |  |  |  |  |  |  |  |  |  |  |  |  |  |  |  |
| **NAH** | 0.137 | 0.000 | 0.000 | 0.000 |  |  |  |  |  |  |  |  |  |  |  |  |  |  |
| **PEL** | 0.103 | 0.000 | 0.019 | 0.010 | 0.000 |  |  |  |  |  |  |  |  |  |  |  |  |  |
| **MXL** | 0.125 | 0.111 | 0.091 | 0.117 | 0.130 | 0.000 |  |  |  |  |  |  |  |  |  |  |  |  |
| **CLM** | 0.368 | 0.395 | 0.402 | 0.415 | 0.420 | 0.112 | 0.000 |  |  |  |  |  |  |  |  |  |  |  |
| **PUR** | 0.366 | 0.385 | 0.390 | 0.405 | 0.411 | 0.113 | 0.005 | 0.000 |  |  |  |  |  |  |  |  |  |  |
| **TSI** | 0.383 | 0.408 | 0.416 | 0.431 | 0.429 | 0.120 | 0.004 | 0.012 | 0.000 |  |  |  |  |  |  |  |  |  |
| **IBS** | 0.369 | 0.394 | 0.402 | 0.418 | 0.416 | 0.115 | 0.001 | 0.008 | 0.000 | 0.000 |  |  |  |  |  |  |  |  |
| **CEU** | 0.375 | 0.397 | 0.403 | 0.418 | 0.419 | 0.119 | 0.002 | 0.011 | 0.000 | 0.001 | 0.000 |  |  |  |  |  |  |  |
| **GBR** | 0.388 | 0.416 | 0.425 | 0.440 | 0.437 | 0.125 | 0.003 | 0.015 | 0.000 | 0.000 | 0.000 | 0.000 |  |  |  |  |  |  |
| **FIN** | 0.383 | 0.410 | 0.418 | 0.431 | 0.432 | 0.130 | 0.018 | 0.030 | 0.027 | 0.022 | 0.012 | 0.017 | 0.000 |  |  |  |  |  |
| **ESN** | 0.331 | 0.354 | 0.361 | 0.373 | 0.378 | 0.177 | 0.187 | 0.150 | 0.204 | 0.195 | 0.196 | 0.215 | 0.206 | 0.000 |  |  |  |  |
| **GWD** | 0.317 | 0.340 | 0.345 | 0.359 | 0.364 | 0.160 | 0.171 | 0.135 | 0.188 | 0.178 | 0.182 | 0.199 | 0.193 | 0.011 | 0.000 |  |  |  |
| **LWK** | 0.301 | 0.319 | 0.324 | 0.335 | 0.345 | 0.150 | 0.161 | 0.131 | 0.182 | 0.173 | 0.174 | 0.192 | 0.181 | 0.011 | 0.015 | 0.000 |  |  |
| **MSL** | 0.313 | 0.337 | 0.343 | 0.357 | 0.363 | 0.168 | 0.189 | 0.159 | 0.209 | 0.199 | 0.201 | 0.220 | 0.211 | 0.010 | 0.011 | 0.009 | 0.000 |  |
| **YRI** | 0.328 | 0.350 | 0.359 | 0.369 | 0.373 | 0.172 | 0.175 | 0.141 | 0.194 | 0.183 | 0.187 | 0.204 | 0.196 | 0.001 | 0.007 | 0.009 | 0.009 | 0.000 |

Supplementary Table 5. Fst values for the I/I genotype from the chromosome 8p23.1 region (1298 SNPs). The darker the colour, the higher the Fst value. Aymara (AYM) were included in the comparisons due to the availability of individuals to compute Fst values.

| **Pop** | **BRS** | **MXL** | **AYM** | **CLM** | **PUR** | **TSI** | **IBS** | **CEU** | **GBR** | **FIN** | **ESN** | **GWD** | **LWK** | **MSL** | **YRI** |
| --- | --- | --- | --- | --- | --- | --- | --- | --- | --- | --- | --- | --- | --- | --- | --- |
| **BRS** | 0.000 |  |  |  |  |  |  |  |  |  |  |  |  |  |  |
| **MXL** | 0.001 | 0.000 |  |  |  |  |  |  |  |  |  |  |  |  |  |
| **AYM** | 0.387 | 0.471 | 0.000 |  |  |  |  |  |  |  |  |  |  |  |  |
| **CLM** | 0.334 | 0.396 | 0.041 | 0.000 |  |  |  |  |  |  |  |  |  |  |  |
| **PUR** | 0.318 | 0.375 | 0.091 | 0.019 | 0.000 |  |  |  |  |  |  |  |  |  |  |
| **TSI** | 0.331 | 0.400 | 0.137 | 0.045 | 0.027 | 0.000 |  |  |  |  |  |  |  |  |  |
| **IBS** | 0.353 | 0.431 | 0.150 | 0.058 | 0.025 | 0.002 | 0.000 |  |  |  |  |  |  |  |  |
| **CEU** | 0.357 | 0.436 | 0.155 | 0.050 | 0.020 | 0.003 | 0.000 | 0.000 |  |  |  |  |  |  |  |
| **GBR** | 0.338 | 0.413 | 0.133 | 0.052 | 0.032 | 0.000 | 0.008 | 0.016 | 0.000 |  |  |  |  |  |  |
| **FIN** | 0.347 | 0.413 | 0.119 | 0.041 | 0.020 | 0.013 | 0.018 | 0.008 | 0.017 | 0.000 |  |  |  |  |  |
| **ESN** | 0.250 | 0.288 | 0.319 | 0.264 | 0.219 | 0.265 | 0.277 | 0.273 | 0.265 | 0.271 | 0.000 |  |  |  |  |
| **GWD** | 0.254 | 0.291 | 0.301 | 0.259 | 0.212 | 0.252 | 0.259 | 0.258 | 0.252 | 0.259 | 0.016 | 0.000 |  |  |  |
| **LWK** | 0.230 | 0.264 | 0.259 | 0.207 | 0.167 | 0.214 | 0.229 | 0.222 | 0.215 | 0.208 | 0.009 | 0.021 | 0.000 |  |  |
| **MSL** | 0.232 | 0.271 | 0.295 | 0.232 | 0.195 | 0.231 | 0.245 | 0.240 | 0.240 | 0.243 | 0.027 | 0.022 | 0.021 | 0.000 |  |
| **YRI** | 0.256 | 0.294 | 0.290 | 0.238 | 0.195 | 0.240 | 0.246 | 0.245 | 0.242 | 0.247 | 0.017 | 0.015 | 0.020 | 0.013 | 0.000 |

Supplementary Table 6. Fst values for the NI/NI and the I/I genotype from the chromosome 8p23.1 region (1298 SNPs), comparing the European tracts only among the Admixed American and Europeans populations. The darker the colour, the higher the Fst value.

| **Genotype** | **Pop** | **BRS.EUR** | **PEL** | **CLM.EUR** | **PUR.EUR** | **TSI** | **IBS** | **CEU** | **GBR** | **FIN** |
| --- | --- | --- | --- | --- | --- | --- | --- | --- | --- | --- |
| **NI/NI** | **BRS.EUR** | 0.000 |  |  |  |  |  |  |  |  |
|  | **PEL** | 0.165 | 0.000 |  |  |  |  |  |  |  |
|  | **CLM.EUR** | 0.407 | 0.443 | 0.000 |  |  |  |  |  |  |
|  | **PUR.EUR** | 0.409 | 0.447 | 0.002 | 0.000 |  |  |  |  |  |
|  | **TSI** | 0.395 | 0.420 | 0.008 | 0.005 | 0.000 |  |  |  |  |
|  | **IBS** | 0.384 | 0.413 | 0.003 | 0.000 | 0.001 | 0.000 |  |  |  |
|  | **CEU** | 0.389 | 0.415 | 0.004 | 0.002 | 0.000 | 0.000 | 0.000 |  |  |
|  | **GBR** | 0.407 | 0.434 | 0.003 | 0.003 | 0.000 | 0.000 | 0.000 | 0.000 |  |
|  | **FIN** | 0.401 | 0.428 | 0.021 | 0.017 | 0.026 | 0.022 | 0.012 | 0.017 | 0.000 |
| **Genotype** | **Pop** | **BRS.EUR** | **MXL.EUR** | **CLM.EUR** | **PUR.EUR** | **TSI** | **IBS** | **CEU** | **GBR** | **FIN** |
| **I/I** | **BRS.EUR** | 0.000 |  |  |  |  |  |  |  |  |
|  | **MXL.EUR** | 0.000 | 0.000 |  |  |  |  |  |  |  |
|  | **CLM.EUR** | 0.347 | 0.401 | 0.000 |  |  |  |  |  |  |
|  | **PUR.EUR** | 0.332 | 0.382 | 0.000 | 0.000 |  |  |  |  |  |
|  | **TSI** | 0.346 | 0.402 | 0.000 | 0.026 | 0.000 |  |  |  |  |
|  | **IBS** | 0.369 | 0.433 | 0.012 | 0.027 | 0.002 | 0.000 |  |  |  |
|  | **CEU** | 0.374 | 0.402 | 0.013 | 0.018 | 0.003 | 0.000 | 0.000 |  |  |
|  | **GBR** | 0.354 | 0.415 | 0.000 | 0.032 | 0.000 | 0.008 | 0.015 | 0.000 |  |
|  | **FIN** | 0.362 | 0.416 | 0.000 | 0.023 | 0.013 | 0.018 | 0.008 | 0.017 | 0.000 |

Supplementary Table 7. Regions with 100-SNP blocks encompassing the highest proportion of outlier SNPs (1% highest FCS values across the genome), which indicates positive selection. The ancestry component of each region was assigned by RFMix.

| **Chr** | **Start–end (bp)** | **Ancestry component** | **Chr** | **Start–end (bp)** | **Ancestry component** |
| --- | --- | --- | --- | --- | --- |
| 1 | 111058469-111370497 | AFR | 14 | 20863811-21182800 | EUR |
| 1 | 18669450-19105313 | NAT | 14 | 22481528-22761250 | AFR |
| 1 | 240611614-241039522 | NAT | 14 | 93866145-94418146 | AFR |
| 3 | 187827820-188260561 | NAT | 15 | 61114403-61369210 | AFR |
| 4 | 7209585-7473205 | NAT | 15 | 98673849-98957608 | NAT |
| 4 | 78226266-78614222 | AFR | 16 | 5908224-6165126 | NAT |
| 5 | 73544647-73823747 | AFR | 16 | 78902324-79126670 | EUR/NAT |
| 5 | 9847219-10150240 | AFR | 16 | 82460189-82680526 | AFR |
| 6 | 6084559-6313384 | NAT | 16 | 85992829-86237547 | AFR |
| 7 | 154928429-155296387 | NAT | 16 | 86460978-86702431 | AFR |
| 7 | 68138053-68549975 | AFR | 17 | 3393962-3978031 | NAT |
| 8 | 1756062-2074042 | EUR/NAT | 17 | 71743780-72169114 | AFR |
| 8 | 4249675-4541063 | EUR | 17 | 76470654-76897475 | EUR |
| 8 | 8964626-9268764 | NAT | 17 | 76901465-77368174 | EUR |
| 9 | 3849117-9148168 | EUR | 18 | 352577-626518 | AFR |
| 9 | 7714889-7997399 | AFR | 20 | 284834-744847 | EUR |
| 9 | 78551222-78843977 | EUR | 21 | 47442576-47476701 | EUR |
| 10 | 12269400-12640797 | EUR | 22 | 26924336-27292868 | NAT |
| 11 | 132043292-132371342 | AFR | 22 | 27453771-27674547 | AFR/EUR |
| 11 | 134680981-134726725 | EUR | 22 | 44362815-44745720 | AFR |
| 11 | 7473121-7784767 | AFR | 22 | 49430916-49691300 | EUR |
| 12 | 129556120-129809027 | NAT |  |  |  |
| 13 | 110714357-110984977 | AFR/NAT |  |  |  |
| 13 | 27268874-27565562 | AFR |  |  |  |
| 13 | 98922590-99298809 | AFR |  |  |  |

Supplementary Table 8. Sample description of BRS sample and data from the1KGP dataset

| **Continental region** | **Abbreviation** | **Population description** | **Size** |
| --- | --- | --- | --- |
| BRS | BRS | Brazilian individuals from São Paulo | 264 |
| AFR | ACB | African Caribbeans in Barbados | 96 |
| AFR | ASW | Americans, African Ancestry in SW USA | 61 |
| AFR | ESN | Esan in Nigeria | 99 |
| AFR | GWD | Gambian in Western Gambia | 113 |
| AFR | LWK | Luhya in Webuye, Kenya | 99 |
| AFR | MSL | Mende in Sierra Leone | 85 |
| AFR | YRI | Yoruba in Ibadan, Nigeria | 108 |
| AMR | CLM | Colombians from Medellin, Colombia | 94 |
| AMR | MXL | Mexican Ancestry from LA, USA | 64 |
| AMR | PEL | Peruvians from Lima, Peru | 85 |
| AMR | PUR | Puerto Ricans from Puerto Rico | 104 |
| EAS | CDX | Chinese Dai in Xishuangbanna, China | 93 |
| EAS | CHB | Han Chinese in Bejing, China | 103 |
| EAS | CHS | Southern Han Chinese | 105 |
| EAS | JPT | Japanese in Tokyo, Japan | 104 |
| EAS | KHV | Kinh in Ho Chi Minh City, Vietnam | 99 |
| EUR | CEU | Utah Residents (CEPH), USA | 99 |
| EUR | FIN | Finnish in Finland | 99 |
| EUR | GBR | British in England and Scotland | 91 |
| EUR | IBS | Iberian Population in Spain | 107 |
| EUR | TSI | Toscani in Italia | 107 |
| SAS | BEB | Bengali from Bangladesh | 86 |
| SAS | GIH | Gujarati Indian from Houston, Texas | 103 |
| SAS | ITU | Indian Telugu from the UK | 102 |
| SAS | PJL | Punjabi from Lahore, Pakistan | 96 |
| SAS | STU | Sri Lankan Tamil from the UK | 102 |

AFR = African; AMR = admixed American; EAS = East Asian; EUR = European; SAS = South Asian.

Supplementary Table 9. Number of SNPs at each filtering step. Step I shows SNP filtering before individual filtering and merging with the1KGP data, and Step II shows SNP filtering after merging with the 1KGP data.

| **Process step** | | **BRS** | **Native-American sample** |
| --- | --- | --- | --- |
| **Step I** | Filter by Autosome SNPs | 868,023 | 862,261 |
|  | Ambiguous SNPs removed | 734,017 | 751,731 |
|  | SNP missing data < 0.02 | 734,017 | 722,422 |
|  | HWE p-value > 0.01 | 605,637 | 719,472 |
| **Step II** | Dataset merging | 599,887* | 573,985** |
|  | MAF > 0.01 from all sample | 596,275 | 570,323 |
|  | LD pruning in all sample | 304,541 | 294,823 |

*BRS merged with 1KGP; **Native-American sample merged with BRS+1KGP.

Supplementary Table 10. Summary results after individual filtering in the BRS dataset.

| **Process step** | **Size (SNP genotyping)** |
| --- | --- |
| Total sample | 264 |
| Individual missing data < 0.02 | 264 |
| Under heterozygosity rate range | 253 |
| IBS < 0.85 | 245 |
| Relatedness < 0.125 | 222 |
